# *In Vitro* Weight-Loaded Cell Models for Understanding Mechanodependent Molecular Pathways Involved in Orthodontic Tooth Movement: A Systematic Review

**DOI:** 10.1155/2018/3208285

**Published:** 2018-07-31

**Authors:** Mila Janjic, Denitsa Docheva, Olivera Trickovic Janjic, Andrea Wichelhaus, Uwe Baumert

**Affiliations:** ^1^Department of Orthodontics and Dentofacial Orthopedics, University Hospital, LMU Munich, 80336 Munich, Germany; ^2^Experimental Trauma Surgery, Department of Trauma Surgery, University Regensburg Medical Centre, 93053 Regensburg, Germany; ^3^Department of Preventive and Pediatric Dentistry, Faculty of Medicine, University of Niš, 18000 Niš, Serbia

## Abstract

Cells from the mesenchymal lineage in the dental area, including but not limited to PDL fibroblasts, osteoblasts, and dental stem cells, are exposed to mechanical stress in physiological (e.g., chewing) and nonphysiological/therapeutic (e.g., orthodontic tooth movement) situations. Close and complex interaction of these different cell types results in the physiological and nonphysiological adaptation of these tissues to mechanical stress. Currently, different *in vitro* loading models are used to investigate the effect of different types of mechanical loading on the stress adaptation of these cell types. We performed a systematic review according to the PRISMA guidelines to identify all studies in the field of dentistry with focus on mechanobiology using *in vitro* loading models applying uniaxial static compressive force. Only studies reporting on cells from the mesenchymal lineage were considered for inclusion. The results are summarized regarding gene expression in relation to force duration and magnitude, and the most significant signaling pathways they take part in are identified using protein-protein interaction networks.

## 1. Introduction

**Figure 1:**
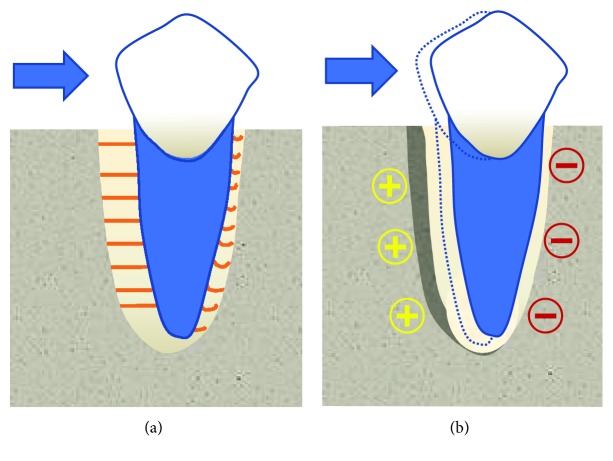
Bone remodelling during orthodontic tooth movement. (a) Initial displacement of the tooth due to stretching of the fibres within the PDL on the tension side and compression on the opposite with the application of the orthodontic force. (b) Bone apposition on the tension side and resorption on the compression side as the result of the long-term force application.

The aim of orthodontics is to move an abnormally positioned tooth through the application of a continuous force on its surface. This force stimulates bone remodelling in the surrounding tissue, namely, the periodontal ligament (PDL) and the alveolar bone, resulting in the bone removal in the direction of the tooth movement and bone apposition in the opposite direction (Figure 1). Thus, the underlying mechanism of orthodontic tooth movement (OTM) is the stimulation of bone remodelling by the application of an orthodontic force [1].

Histologically, the effects of orthodontic force on the tooth and its surrounding tissues are now well understood and the underlying stages in OTM are identified [2]. Human periodontal ligament cells (hPDLCs) and human osteoblasts (hOBs) are recognized as the cell types originating from the mesenchymal lineage, which play the most dominant role during OTM. Unlike hOBs, which represent well a characterized cell type, hPDLCs represent a mixed population of mostly fibroblast-like cells [3]. Among them, mesenchymal stem cells are of special importance as the source of progenitors responsible for the regeneration and remodulation of not only PDL itself but also alveolar bone [4].

In order to better understand morphological changes during OTM, it is important to elucidate molecular and cellular signaling mechanisms between and within these cell types. The complex *in vivo* structure of the tissues involved makes it impossible to investigate force sensing and cellular communication of individual cells. Therefore, *in vitro* models using cells isolated from the PDL or from alveolar bone were established and different types of forces mimicking those found during OTM were applied [5]. These *in vitro* models are used to answer open questions including but not limited to how cells sense force, how they convert mechanical stress into molecular signals, and how these molecular signals influence the specific response of these cells to that specific force.

On the basis of the most commonly used approaches to apply mechanical stress on cells, present *in vitro* loading models can be classified into those using substrate deformation-based approaches, hydrostatic pressure approach, centrifugation approach, fluid flow approach, vibration approach, and weight approach [6]. Also, there has been increasing interest in moving from conventional monolayer, two-dimensional (2D) *in vitro* loading models to three-dimensional (3D) *in vitro* loading models.

**Figure 2:**
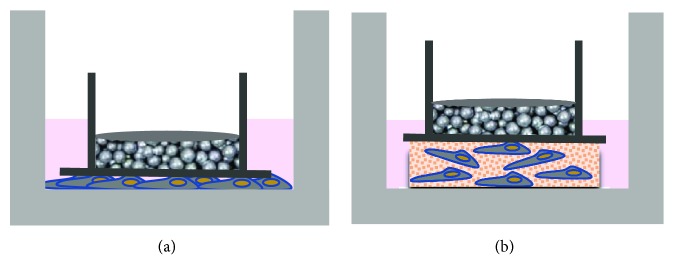
Schematic illustration of the static 2D (a) and 3D (b) *in vitro* loading model based on the weight approach applied in the literature (details are found in the text).

Weight-based *in vitro* loading models have been successfully used over several years to investigate the effect of static, compressive, unidirectional force on the cells. In models using 2D cell cultures, cells are precultured in cell culture dishes (e.g., 6-well plates). After reaching the desired confluency, the cells are subjected to weight-based compression. In most cases, a glass slide is laid on top of the cell monolayer. Then, a weight is applied by positioning a glass cylinder filled with lead granules on top of this slide. The glass slide is used to secure even distribution of the force [7]. Increasing or reducing the number of granules in the glass cylinder adjusts the level of compressive force (Figure 2(a)). The same type of force is applied by slight modifications of this model: some authors used a stack of glass slides of different heights (e.g., [8]) or glass discs of different thicknesses (e.g., [9]) replacing the glass cylinder filled with lead granules. This *in vitro* loading model can also be used to apply static compressive force on 3D cell cultures. In this case, the same principle is used, except that the cells are embedded in a 3D matrix that is then compressed in the described manner (Figure 2(b)). Yang et al. [6] coined the term “weight approach”-based (WAB) for this *in vitro* model. To refer to this specific setup, we will also use WAB throughout this publication.

The primary aim of this review was to identify all articles related to the field of orthodontics using either a 2D or 3D WAB *in vitro* loading model and provide an overview of the details of their use: the most commonly used loading durations, force magnitudes, and scaffolds and their findings regarding gene expression and substance secretion in relation to force application. The secondary objective was to discover most commonly examined genes and to identify important pathways in OTM that most of the identified genes from these studies are involved in, focusing especially on hPDLCs.

## 2. Materials and Methods

To conduct this review, the “Preferred Reporting Items for Systematic Review and Meta-Analysis Protocols” (PRISMA-P) 2015 statement was consulted [10].

### 2.1. Defining the Eligibility Criteria

Inclusion criteria were as follows:

- Studies in the field of dentistry that examined the effect of mechanical stress on tooth surrounding tissues
- Application of the 2D or 3D WAB *in vitro* loading model…
- …on hPDLCs, hOBs, or all bone-like cell types/lines of human or animal origin
- Only studies written in English language, identified on the PubMed database until 01.12.2017, were taken into consideration

### 2.2. Literature Search and Study Selection Process

Separate search strategies were created for studies using either the 2D or the 3D *in vitro* setup for mechanical cell loading (Supplement 1). Searches were performed in the PubMed database following these predefined search strategies.

After identification of relevant studies in the PubMed database, the downloaded records from each search were imported into the bibliographic software EndNote X8 (Clarivate Analytics, Philadelphia, Pennsylvania, USA). All records were examined by two reviewers independently (MJ and UB), according to predefined inclusion and exclusion criteria (see above): first by title, then by abstract. If the abstract was not available, the full text of the report was obtained. Records that were obviously irrelevant were excluded, and the full texts of all remaining records were acquired. After the full-text assessment, the final list of included articles was generated. Any disagreements during this process were dissolved through discussion with another review author (DD) until reaching a consensus. The articles that did not meet all inclusion criteria after full-text assessment were excluded from further examination. Additional relevant studies were further identified through forward and backward reference chaining and hand-search of specific journals. Study quality assessment of the included studies was not performed, since the goal of this article was to provide an overview of all findings in the field only.

### 2.3. Data Extraction

The following information was extracted from each study obtained in full length: author, journal, year of publication, and used cell type. Force magnitude and duration, examined genes or substances, gene expression, or substance secretion details were recorded only if their response was directly connected to mechanical force stimulus. Gene symbols were used in the tables whenever possible. In case the identity or variant of a gene was doubtful or not clear primer sequences were examined using Primer-BLAST (URL: https://www.ncbi.nlm.nih.gov/tools/primer-blast/) [11]. If Western blot, ELISA, or inhibition experiments were reported, we tried to verify the antibodies and/or inhibitor specificity to determine the exact protein species (variant). Additionally, the method used for evaluation of the gene/substance expression was recorded. Data regarding the used scaffolds were collected for studies applying 3D WAB *in vitro* setups.

The following tables were prepared to summarize the findings: (1) studies applying the 2D WAB *in vitro* loading model on human primary cells from the orofacial region (i.e., hPDLCs, hOBs, and human oral bone marrow cells), (2) studies applying the 2D WAB *in vitro* loading model on human and nonhuman cells and cell lines not included in the first table, and (3) studies applying the 3D WAB *in vitro* loading model on human and nonhuman cells and cell lines.

### 2.4. STRING Analysis

The examined genes and metabolites using the 2D approach were summarized in two separate lists: one for hPDLFs and one for hOBs and other human bone-derived cell lines. Protein-protein interaction (PPI) networks were generated for both lists separately using the STRING database (10.5, URL: https://string-db.org/) [12]. From within STRING, the KEGG database [13] was queried to identify the main pathways involved. Only pathways with a false discovery rate below 1.00*E*−05 were considered.

## 3. Results

### 3.1. Study Selection Process

**Figure 3:**
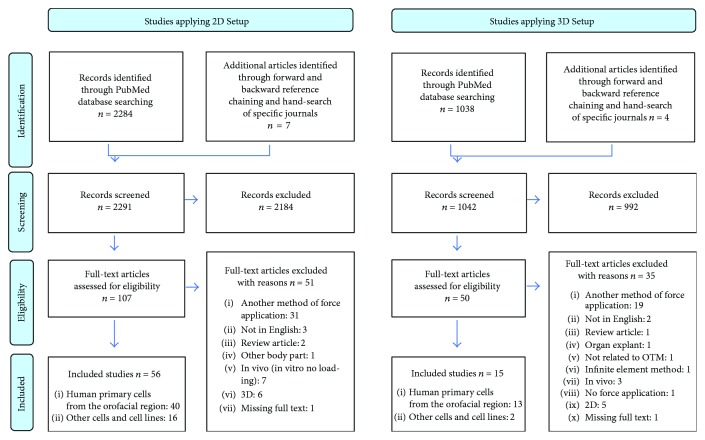
PRISMA flow diagram of the review process.

Figure 3 summarises the results of both 2D and 3D searches using a flow chart according to PRISMA. Separate searches were conducted for the studies applying either the 2D or 3D (Supplement 1) WAB *in vitro* loading models.

The search formula applied to identify 2D WAB *in vitro* loading studies is shown in Supplement 1. Altogether, 2284 abstracts were identified in the PubMed database (Figure 3).

Additionally, 7 articles were identified through forward and backward reference chaining and hand-search of specific journals. After reading the titles and abstracts of all identified studies, we excluded 2184. The remaining 107 articles were then checked by full-text reading. Fifty-six of them meet our inclusion criteria and were included for further analysis. The remaining did not meet the inclusion criteria. Reasons for their exclusion are listed in Supplement 1.

The search formula applied to identify 3D WAB *in vitro* loading studies is shown in Supplement 1. We identified a total of 1038 articles in PubMed (Figure 3). Additional 4 articles were discovered through forward and backward reference chaining and hand-search of specific journals. After initial screening, we excluded 992 articles and proceeded with full-text reading of the 50 articles. Finally, 17 of them meet our inclusion criteria. The remaining articles were excluded from further analysis. Reasons for their exclusion are summarized in Supplement 1.

All studies fulfilling the inclusion criteria were organised into three different supplementary tables: Supplement 2 summarises 2D WAB *in vitro* loading studies using human primary cells from the orofacial region. In Supplement 3, the two-dimensional WAB *in vitro* loading studies using human nonorofacial-derived cells and animal cells and cell lines are found.  Supplement 4 summarises the 3D WAB *in vitro* loading studies.

### 3.2. Force Durations and Force Magnitudes Used in the Studies

#### 3.2.1. 2D WAB *In Vitro* Loading Model

In these studies, compression forces ranging from 0.25 g/cm^2^ to 5 g/cm^2^ were applied on cells in 2D culture. The most commonly used compressive force was 2 g/cm^2^, irrespectively which cell type was used in the study. In most of the studies, the force was applied for 24 h (Supplements 2 and 3).

#### 3.2.2. 3D WAB *In Vitro* Loading Model

Force duration and magnitude depended on the scaffold used (Supplement 4). In most of the studies, scaffolds made from collagen gel and the polylactic-co-glycolic acid (PLGA) were applied. One of the studies [14] used a hydrophilically modified poly-L-lactide (PLLA) matrix. Collagen gel scaffolds were used with force magnitudes varying between 0.5 g/cm^2^ and 9.5 g/cm^2^; the most commonly used force was 6 g/cm^2^. Force was applied for 0.5 to 72 h. Most commonly used force application periods were 12 and 24 h. Force levels between 5 and 35 g/cm^2^ were applied to cells embedded in PLGA scaffolds. The most commonly applied force was 25 g/cm^2^. The duration of force application was from 3 to 72 h. The study using the hydrophilically modulated PLLA matrix [14] applied force magnitudes from 5 to 35 g/cm^2^. The duration of force application varied between one day and 14 days.

### 3.3. Cell Types Used in the Studies

#### 3.3.1. 2D WAB *In Vitro* Loading Model

Forty of these studies used human primary cells isolated from the tooth surrounding tissues (Supplement 2): hPDLCs, hOBs, and human orofacial bone marrow-derived cells (hOBMC). The remaining studies used other cells and cell lines from human and animal sources: MG63, RAW264.7, ST-2, Saos-2, OCCM-30, MC3T3-E1, C2C12, U2OS, rat-derived PDLCs, or bone marrow-derived osteoblasts and the cementoblast cell line HCEM-SV40 (Supplement 3).

#### 3.3.2. 3D WAB *In Vitro* Loading Model

hPDLCs and human gingival fibroblasts were used in 13 studies (Supplement 4). The remaining two studies used cell types and lines from the nonoral region or nonhuman origin (Supplement 4): the murine cell line MC3T3-E1 and murine osteoblasts.

Taken together, the most commonly used cells were hPDLCs. They were used in total 51 studies (2D: 38; 3D: 13) (Supplements 2 and 4). According to the isolation method applied, we distinguished between the following sources: “explant method” [15, 16] (2D: 18; 3D: 4), “enzyme digestion method” [4] (2D: 9; 3D: 6), commercial sources (2D: 3; 3D: 1), or “no detailed information of isolation available” (2D: 8; 3D: 2).

### 3.4. Genes and Substances Examined in the Studies

A complete overview of genes and metabolites examined in 2D and 3D WAB studies and details of their expression can be found in Supplements 2 and 3 (2D) and Supplement 4 (3D).

In this review, special attention was paid to hPDLCs as the most examined cell type among studies and their prominent role in OTM. The most examined genes and metabolites in relation to hPDLCs were TNF superfamily member 11 (TNFSF11), TNF receptor superfamily member 11B (TNFRSF11B), prostaglandin-endoperoxide synthase 2 (PTGS2), and prostaglandin E_2_ (PGE_2_). In Table 1, details regarding their expression/secretion, including the information at which time points or force magnitudes the highest/lowest value was reached, is summarized.

### 3.5. STRING Analysis and KEGG Pathways

#### 3.5.1. Construction of Protein-Protein Interaction (PPI) Network

**Figure 4:**
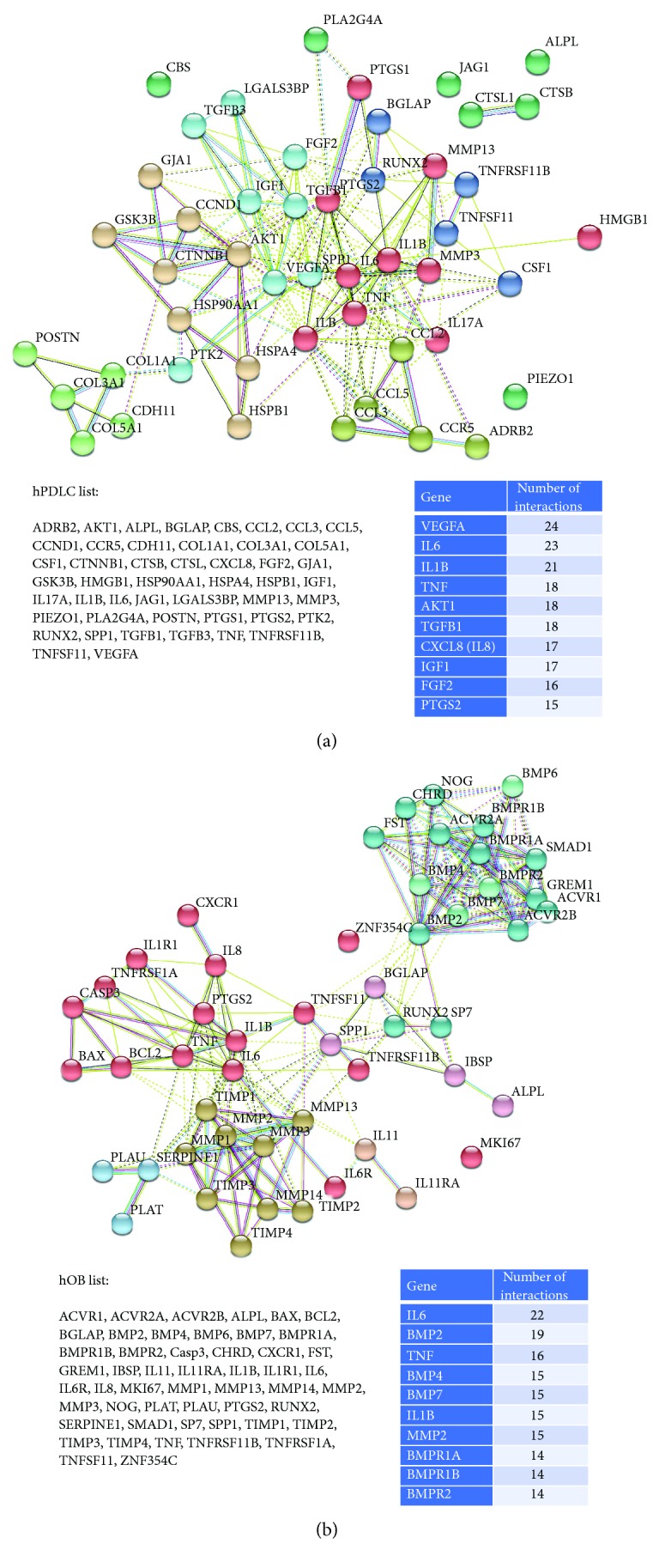
Protein-protein interaction networks for the (a) “hPDLC list” and the (b) “hOB list”. The gene lists are shown in the lower left part of each subfigure. Those genes with the highest number of interactions (“top 10”) are given in tables in the lower right part of each subfigure.

In order to elucidate the molecular mechanisms of OTM and the role of the hPDLCs and bone cells in this process, we used STRING to construct PPI networks. Two separate gene lists were compiled from those studies using hPDLCs (“hPDLC list”; data from Supplement 3) and from those using hOBs or human bone-cells and cell lines (“hOB list”; data from Supplements 2 and 3). The hPDLC list contained 48 different genes (Figure 4(a)) and the hOB list 51 different genes (Figure 4(b)).

Two separate PPI networks were obtained, based on the interactions with a high level of confidence (>0.700) (Figure 4). Nodes in the networks represent the proteins produced by a single protein-coding gene locus; edges represent protein-protein interaction. Based on the colour of the edge, eight different interactions based on “gene neighbourhood,” “gene fusion,” “cooccurrence,” “coexpression,” “experiments,” “databases,” and “text mining” can be differentiated [12]. The top 10 nodes with the highest degree of connections from each of the two gene lists are also shown in Figure 4. PPI enrichment *p* values for each constructed network were calculated in STRING. These show that both PPI networks had significantly more interactions than expected and that the nodes are not random (PP enrichment *p* value < 1.0*E*–16).

#### 3.5.2. Identification of KEGG Pathways

According to our STRING analysis, KEGG pathways relevant for OTM for each set of genes are listed in Table 2.

## 4. Discussion

*In vivo* bone remodelling during OTM represents a complex biological process, triggered by mechanical stimuli. OTM involves numerous events, spatially and temporary orchestrated and coordinated by different cell types, signaling factors, and networks [1]. Systematic breakdown and analysis of individual components of this complex process is the key for understanding its molecular background and a possible way to accelerate and improve it. Therefore, a variety of *in vitro* mechanical loading models have been established [5, 6]. The *in vitro* loading model based on the weight approach has been considered as the most appropriate loading model for the stimulation of the orthodontic force on the compressive site [6].

### 4.1. Characteristics of 2D and 3D WAB *In Vitro* Loading Models

#### 4.1.1. Conventional 2D WAB

In vitro *loading model*, initially described by Kanai et al. [7], has been used for more than two decades for studying the compression-induced osteoclastogenesis and is still considered as the gold standard. It represents a simple and effective method for application of static compressive, unidirectional force to a cell monolayer.

The advantages of WAB *in vitro* loading model are the following:

- It reduces the need for animal studies, which are costly and time consuming.
- It enables the analysis of specific cell types independently or in cocultures with other cells of interest.
- Human primary cells can be used for better approximation to clinical situation.

From our point of view, the main disadvantage is its missing impact of the natural surrounding environment. There has been an increasing interest in the development of the 3D cell culture WAB *in vitro* loading model during the last years, in order to approximate the *in vitro* situation to the *in vivo* situation.

#### 4.1.2. 3D WAB *In Vitro* Loading Model

During the last years, more studies have been using cells incorporated into biological scaffolds instead of monolayer cultures. This is due to the demand of mimicking an extracellular matrix, which is beneficial for cell behaviour, instead of growing cells on artificial plastic cell culture surface [46]. According to our data, three types of scaffolds have been used so far in combination with the 3D WAB *in vitro* loading model. The first identified studies used collagen I scaffolds [26, 47, 48]. Although the collagen gels are still widely used for this purpose, there is the increasing interest in the development of scaffolds composed of synthetic polymers. In 2011, Li et al. [33] introduced the PLGA scaffolds that had a higher stiffness in comparison to collagen gels and an elastic modulus very close to that of human PDL. The only disadvantage was that cells growing in PLGA displayed a disordered grow pattern that differs from the one in natural PDL [33]. Liao et al. [14] went one step further and introduced a hydrophilically modified PLLA matrix. This matrix displayed several advantages: higher nutrient and oxygen permeability and a better cell attachment, making it more suitable for long-term force application [14].

### 4.2. Force Magnitude Used in the Studies

According to Schwarz [49], optimal orthodontic force (OOF) in clinical orthodontics should be equal to capillary blood vessel pressure (≈25 g/cm^2^) [49]. On a tissue level, OOF should enable the desired clinical outcome without causing the unwanted side effects, for example, root resorption. On the cellular level, it should evoke best biologic cellular response without inhibiting the cell proliferation significantly [27]. Optimal orthodontic force *in vitro* varies between different models. Estimation of OOF for each *in vitro* model is of crucial importance for their successful application in OTM simulation [20, 33].

In 2D cell culture WAB *in vitro* loading models, applied forces varied between 0.2 and 5.0 g/cm^2^. Our data suggest that 2.0 g/cm^2^ was the most commonly used force magnitude in the studies so far. According to Kanzaki et al. [20], this force magnitude proved to induce the best cellular response. Few studies reported a decrease in cell viability in a force-dependent manner, especially with the application of 4 g/cm^2^ force [20, 37, 50, 51].

In studies applying the 3D WAB *in vitro* loading models, the force magnitude used was chosen depending on the stiffness of the scaffold. Studies using collagen gel scaffolds most commonly applied 6 g/cm^2^ force onto their *in vitro* models. According to Araujo et al. [47], this force was corresponding to the therapeutic orthodontic force, giving the best cellular response. For PLGA scaffolds, the force magnitude showing the best performance was 25 g/cm^2^ (range: 5–35 g/cm^2^). The same range of forces were applied in the study of Liao et al. [14] using a hydrophilically modified PLLA scaffold matrix. This range also corresponds to the one used in clinical settings, which indicates that these scaffolds are closest to the mechanical properties of *in vivo* PDL [14, 33]. This qualifies them also as a suitable model for investigation of light and heavy forces, which are considered as a cause of orthodontic treatment failure.

### 4.3. Duration of the Force Application

The length of the force application in the studies rarely exceeded 72 h. In most of the cases, force was applied up to 24 and 48 h. Considering the fact that the first 10 days are of crucial importance for OTM ([52], p. 303), the duration of force application in most of the conducted studies is insufficient to fully understand the molecular background of OTM. Additionally, we would like to point out that only a few studies observed cell viability during the experiment. Most of them confirmed a reduction of cell viability, not only due to the force level but also depending on time [19, 50, 51]. We assume that one of the limitations, especially in the 2D WAB *in vitro* models, is compromised nutrient and oxygen supply in the pressure area. To overcome especially the time limitation of previous models, Liao et al. [14] introduced the hydrophilically modified PLLA matrix as a new scaffold for 3D cultures. They have shown that this scaffold can be used for up to 14 days without affecting cell viability, claiming that it provides good perfusion of the nutrients and oxygen over longer periods of time [14]. Establishing an *in vitro* model suitable for long-term force application (up to or more than 10 days) is beneficial for progress in this research field.

### 4.4. Role of PDL and hPDLCs in OTM

Due to lack of PDL, ankylosed teeth and implants cannot undergo OTM, which depict best PDL's key role in transmitting the mechanical stimulus and initiating the process of bone remodelling [1, 53]. Beside its mechanotransduction properties, it also contributes to tissue homoeostasis and repair, mostly due to the presence of mesenchymal stem cells which are an important part in the normal hPDLC population [4]. This portion of hPDLCs is known to be present in a higher extent in hPDLCs isolated with the “enzyme digestion method” [54], commonly used among the studies in this review, especially in the 3D group.

### 4.5. Most Examined Genes in the Studies That Used hPDLCs

To explain the contribution of hPDLCs in OTM on the molecular level, we summarised all data regarding the most commonly examined genes and substances in this cell type (Table 1). These were *TNFSF11*, *PTGS2*, and PGE_2_, known as osteoclastogenesis inducers, and *TNFRSF11B*, known as an osteoclastogenesis inhibitor.

*TNFSF11* (also known as “RANKL”) [55] plays a crucial role in bone resorption on the compression side during OTM, inducing the osteoclast formation. *TNFSF11* showed an increased gene expression in all studies that used the 2D WAB *in vitro* loading model (Table 1). In most of the studies using this model, *TNFSF11* gene expression, as well as protein secretion, was positively correlated with both force duration and magnitude reaching the maximum expression level after 12–24 hours of force application. Studies using the 3D WAB *in vitro* loading model also reported an increase in the TNFSF11 secretion, most of them after 6 hours of force application (Table 1). In cells grown in PLGA scaffolds, a positive correlation between force magnitude and gene expression but a negative correlation between force duration and gene expression was noticed.

*TNFRSF11B*, also referred to as osteoprotegerin (OPG), is TNFSF11's antagonist that inhibits osteoclastogenesis [55]. Most of the studies applying the 2D WAB *in vitro* loading model reported no observed change in gene expression (*n* = 8), with exception of two studies that reported downregulation [40] or transitory downregulation [8] (Table 1). Considering protein secretion, results were contradictory. Most studies, however, reported a decrease in protein secretion or did not report any change. Results from studies using 3D WAB *in vitro* loading were also contrary, depending on the scaffold used. In a study using collagen gel scaffolds, an increase in *TNFRSF11B* gene expression was observed [26]. In all studies applying PLGA scaffolds, a decrease in TNFRSF11B secretion was positively correlated with force magnitude and negatively correlated with force duration [27, 28, 31, 33, 43]. With one exception [28], a comparison of *TNFSF11* and *TNFRSF11B* gene expression in the aforementioned studies showed that a rapid down/regulation of *TNFRSF11B* appears parallel to a rapid upregulation of *TNFSF11* in 3D WAB *in vitro* loading. Since both genes represent antagonists in bone turnover regulation, this was explained as a good representation of the cyclic changes in the bone metabolism on the compression side during OTM [31, 33]. It was also suggested that downregulation of *TNFSF11* in later stages might have something to do with other inducers for prolonged osteoclastogenesis promotion [33].

Gene expression of *PTGS2* was increased upon force application in both 2D and 3D studies. In most of the 2D WAB studies, *PTGS2* showed a positive correlation between the duration of the experiment and gene expression (Table 1). In those studies, using the 3D WAB *in vitro* loading model, *PTGS2* seemed to be negatively correlated with force duration and positively correlated with force magnitude. On the other hand, PTGS2 protein quantity was shown to be in positive correlation with both duration and force magnitude using Western blotting (Table 1). Since PTGS2 is involved in prostaglandin E_2_ metabolism, an upregulation of *PTGS2* gene expression (maximum at 24 to 48 h after force application) is correlated with an upregulation of PGE_2_ secretion (maximum at 48 h after force application) in all studies (Table 1).

Taken together, there seems to be some inconsistency between studies using the 2D and the 3D WAB *in vitro* loading model. The results within the 2D WAB group of studies are quite similar and comparable. However, a noticeable higher heterogeneity among those studies using the 3D WAB *in vitro* loading model is recognizable. This heterogeneity can be related to the type of scaffolds used.

### 4.6. STRING PPI Analysis

We performed STRING PPI analysis for two selected sets of genes (“hPDLC list” and “hOB list”). PPI enrichment *p* values obtained from both PPI networks (Figure 4) had significantly more interactions than expected. This implicates that the genes examined in the studies were not chosen randomly. From our point of view, this is not surprising, since most of the studies were selecting “the genes of interest” for their analysis, all previously known or suspected to be involved in bone metabolism. Just a few of the studies performed microarray analysis in order to identify all genes responding to force application [26, 32, 44, 48].

In addition, KEGG pathways relevant for OTM, identified for each set of genes in STRING analysis (Table 2), can be useful source for discovering new genes that might influence OTM.

## 5. Conclusions

In summary, the WAB *in vitro* loading model represents a simple and very efficient way to investigate molecular events during OTM. The purpose of this review was to provide an overview of all used forms of the WAB *in vitro* loading model (2D and 3D in combination with different scaffolds), present all current findings, and point out at certain questions for their further improvement.

3D WAB *in vitro* loading models have shown to be promising for use in future research by bringing a more real environment in *in vitro* setups. However, unlike well-established 2D models that provide comparable results, 3D models show inconsistency in results. Obviously, there is a need for further improvement in order to establish standardised *in vitro* models that will provide comparable results. Also, there is a need to elucidate molecular events during longer periods of force application. Therefore, the future goal is to establish both 2D and 3D loading models that will allow us to conduct long-term investigations. The study of Liao et al. [14] is a good example for this, and there should be more research in that direction.

## Figures and Tables

**Table 1:** Top four examined genes or substances in studies applying 2D or 3D *in vitro* WAB loading model on hPDLCs. For each gene or substance, cell culture type and literature reference are given. Additionally, examined force durations and magnitudes are summarized. Force effect on gene expression/substance secretion was evaluated (increase/decrease/no change). In all cases, the most prominent changes (increase/decrease) or “no change” are noted. For each change of expression/secretion, the corresponding maximum (increase/decrease) of force duration and magnitudes are additionally provided.

Gene symbol or metabolite	Cell culture	Reference	Examined force applied		Gene expression			Substance secretion		
			Duration (h)	Magnitude (g/cm^2^)	Increase/decrease/no change	Change in relation to force duration (h)	Change in relation to force magnitude (g/cm^2^)	Increase/decrease/no change	Change in relation to force duration (h)	Change in relation to force magnitude (g/cm^2^)
PGE_2_	2D	Benjakul et al. in press [17]	48	1.5	na			Increase (qPCR: GAPDH)	48	1.5
		Jin et al. 2015 [18]	0; 0.5; 3; 6; 12	2.0	na			Increase (ELISA)	12	2.0
		Kang et al. 2010 [19]	0.5; 2; 6; 24; 48	2.0	na			Increase (ELISA)	48	2.0
		Kanzaki et al. 2002 [20]	0.5; 1.5; 6; 24; 48 (+ELISA: 60)	0.5; 1.0; 2.0; 3.0; 4.0 (ELISA: 2.0)	na			Increase (ELISA)	60	2.0
		Kirschneck et al. 2015 [21]	24	2.0	na			Not explicitly stated (ELISA)		
		Liu et al. 2006 [22]	48	2.0	na			Increase (ELISA)	48	2.0
		Mayahara et al. 2007 [23]	3; 6; 12; 24; 48	2	na			Increase (ELISA)	48	2
		Premaraj et al. 2013 [24]	0.5; 1; 3; 6	5.0	na			Increase (ELISA)	1	5.0
		Proff et al. 2014 [9]	24	2	na			Increase (ELISA)	24	2
		Römer et al. 2013 [25]	24	2	na			Increase (ELISA)	24	2
	3D (Coll. gel)	de Araujo et al. 2007 [26]	3; 12; 24; 48; 72	6.0				Increase (EIA)	72	6.0
	3D (PLGA)	Li et al. 2016 [27]	6; 24; 72	5.0; 15.0; 25.0	na			Increase (ELISA)	24	15.0…25.0
		Yi et al. 2016 [28]	24	25.0				Increase (ELISA)	24	25.0

*PTGS2*	2D	Jin et al. 2015 [18]	0; 0.5; 3; 6; 12	2.0	Increase (qPCR: GAPDH)	12	2.0			
		Kang et al. 2010 [19]	0.5; 2; 6; 24; 48	2.0	Increase (qPCR: GAPDH)	48				
		Kanzaki et al. 2002 [20]	0.5; 1.5; 6; 24; 48	0.5; 1.0; 2.0; 3.0; 4.0	Increase (sqPCR: ACTNB)	6	2.0			
		Kirschneck et al. 2015 [21]	24	2.0	Increase (qPCR: POL2RA)	24	2.0			
		Liu et al. 2006 [22]	48	2.0	Increase (sqPCR: ACTNB)	48	2.0			
		Mayahara et al. 2007 [23]	3; 6; 12; 24; 48	2	Increase (qPCR: GAPDH)	48	2			
		Mayahara et al. 2010 [29]	3; 6; 12; 24; 48	2.0	Increase (qPCR: GAPDH)	48	2			
		Premaraj et al. 2013 [24]	6	0.2; 2.2; 5.0	nd			Increase (WB)	6	5.0
		Proff et al. 2014 [9]	24	2	Increase (qPCR: POL2RA)	24	2	Increase (WB)	24	2
		Römer et al. 2013 [25]	24	2	Increase (qPCR: POL2RA)	24	2			
		Wongkhantee et al. 2007 [30]	24	0; 1.25; 2.5	Increase (sqPCR: GAPDH)	24	2.5			
	3D (Coll. gel)	de Araujo et al. 2007 [26]	1; 3; 6; 12; 24; 48; 72	3.6; 6.0; 7.1; 9.5	Increase (sqPCR: GAPDH)	6	7.1			
	3D (PLGA)	Li et al. 2016 [31]	6; 24; 72	25.0	Increase (qPCR: GAPDH)	6	25.0			
		Li et al. 2013 [32]	6; 24; 72	25.0	Increase (qPCR: GAPDH)	6	25.0			
		Li et al. 2016 [27]	6; 24; 72	5.0; 15.0; 25.0	Increase (qPCR: GAPDH)	6	25.0			
		Li et al. 2011 [33]	6	5; 15; 25; 35	Increase (qPCR: GAPDH)	6	35.0			
		Yi et al. 2016 [28]	24	25.0	Increase (qPCR: GAPDH)	24	25.0	Increase (WB)	24	25.0

*TNFRSF11B*	2D	Benjakul et al. in press [17]	48	1.5	No change (qPCR: GAPDH)			No change		
		Jin et al. 2015 [18]	0; 0.5; 3; 6; 12	2.0	No change (qPCR: GAPDH)					
		Kanzaki et al. 2002 [20]	0.5; 1.5; 6; 24; 48	0.5; 1.0; 2.0; 3.0; 4.0	No change (sqPCR: ACTNB)					
		Kim et al. 2013 [8]	0.5; 2; 6; 24; 48	2.0	Transitory downregulated. (qPCR: GAPDH)	6	2.0	Transitory downregulation (ELISA)	6	2.0
		Kirschneck et al. 2015 [21]	24	2.0	No change (qPCR: POL2RA)					
		Lee et al. 2015 [34]	0; 2; 4; 8; 24; 48	2.5	No change (qPCR: ACTNB)					
		Liu et al. 2017 [35]	6; 12; 24	0.5; 1.0; 1.5	nd			Decrease (WB)	n. g.	1.5
		Luckprom et al. 2011 [36]	2; 4	2.5	No change (sqPCR: GAPDH)					
		Mitsuhashi et al. 2011 [37]	1; 3; 6; 9; 12; 24	4.0	No change (qPCR: ACTNB)					
		Nakajima et al. 2008 [38]	0; 1; 3; 6; 9; 12; 24	0.5; 1.0; 2.0; 3.0; 4.0	nd			Increase (ELISA)	24	0.5
		Nishijima et al. 2006 [39]	48	0; 0.5; 1.0; 2.0; 3.0	nd			Decrease (ELISA)	48	2.0
		Römer et al. 2013 [25]	24	2	No change (qPCR: RNA-polymerase-2-polypeptide A)					
		Yamada et al. 2013 [40]	12	4.0	Decrease (qPCR: GAPDH)	12	4.0	Decrease (ELISA)	12	4.0
		Yamaguchi et al. 2006 [41]	0; 3; 6; 9; 12; 24; 48	0.5; 1.0; 2.0; 3.0	n. d.			Decrease (ELISA)	12…48	2.0
	3D (Coll. gel)	Kaku et al. 2016 [42]	12; 24	0.5; 1.0; 2.0	Increase (qPCR: GAPDH)	12	1.0			
	3D (PLLA modif.)	Liao et al. 2016 [14]	1 d; 3 d; 7 d; 14 d	5.0; 15.0; 25.0; 35.0	No change (qPCR: GAPDH)					
	3D (PLGA)	Jianru et al. 2015 [43]	3; 6; 12 (WB: 12)	25.0	Decrease followed by increase (qPCR: GAPDH)	3 (decrease) 12 (increase)	25.0	Increase (WB)	12	25.0
		Li et al. 2016 [31]	6; 24; 72	25.0	Decrease followed by Increase (qPCR: GAPDH)	6 (decrease) 72 (increase)	25.0			
		Li et al. 2016 [27]	6; 24; 72	5.0; 15.0; 25.0	Decrease followed by increase (qPCR: GAPDH)	6 (decrease) 72 (increase)	15.0 (decrease) 25.0 (increase)	Decrease followed by Increase (qPCR: GAPDH)	6 (decrease) 72 (increase)	25.0 (decrease) 25.0 (increase)
		Li et al. 2011 [33]	6; 24; 72	25	Decrease followed by increase (qPCR: GAPDH)	6 (decrease) 72 (increase)	25.0			
		Yi et al. 2016 [28]	24	25.0	Decrease (qPCR: GAPDH)	24	25.0	No change (WB)		

*TNFSF11*	2D	Benjakul et al. in press [17]	48	1.5	Increase (qPCR: GAPDH)	48	1.5	Increase (qPCR: GAPDH)	48	1.5
		Jin et al. 2015 [18]	0; 0.5; 3; 6; 12	2.0	Increase (qPCR: GAPDH)	12	2.0			
		Kang et al. 2013 [44]	2; 48	2.0	Increase (qPCR: GAPDH)	48	2.0			
		Kanzaki et al. 2002 [20]	0.5; 1.5; 6; 24; 48	0.5; 1.0; 2.0; 3.0; 4.0	Increase (sqPCR: ACTNB)	48	2.0	Increase (WB): 40-kDa+ 55-kDa	48	2.0
		Kikuta et al. 2015 [45]	1; 3; 6; 9; 12; 24 (+ELISA: 48)	4.0	Increase (qPCR: GAPDH)	12	4.0	Increase (ELISA)	24	4.0
		Kim et al. 2013 [8]	0.5; 2; 6; 24; 48	2.0 ++	Increase (qPCR: GAPDH)	24	2.0	Increase (ELISA)	48	2.0
		Kirschneck et al. 2015 [21]	24	2.0	Increase (qPCR: POL2RA)	24	2.0			
		Lee et al. 2015 [34]	0; 2; 4; 8; 24; 48	2.5	Increase (qPCR: ACTNB)	24	2.5			
		Liu et al. 2017 [35]	6, 12, 24	0.5; 1.0; 1.5	nd			Increase (WB: GAPDH)	ng	1.5
		Liu et al. 2006 [22]	48	2.0	Increase (sqPCR: ACTNB)	48	2.0			
		Luckprom et al. 2011 [36]	2; 4	2.5	Increase (sqPCR: GAPDH)	2	2.5	Increase (WB)	4	2.5
		Mitsuhashi et al. 2011 [37]	1; 3; 6; 9; 12; 24	4.0	Temporary increase (qPCR: ACTNB)	6…9	4.0			
		Nakajima et al. 2008 [38]	0; 1; 3; 6; 9; 12; 24	0.5; 1.0; 2.0; 3.0; 4.0	nd			Increase (ELISA)	24	4.0
		Nishijima et al. 2006 [39]	48	0; 0.5; 1.0; 2.0; 3.0	nd			Increase (ELISA)	12…48	2.0
		Römer et al. 2013 [25]	24	2	Increase (qPCR: RNA-polymerase-2-polypeptide A)	24	2			
		Wongkhantee et al. 2007 [30]	24	0; 1.25; 2.5	Increase (sqPCR: GAPDH)	24	2.5	Increase (WB; ACTNB)	24	2.5
		Yamada et al. 2013 [40]	12	4.0	Increase (qPCR: GAPDH)	12	4.0	Increase (ELISA)	12	4.0
		Yamaguchi et al. 2006 [41]	0; 3; 6; 9; 12; 24; 48	0.5; 1.0; 2.0; 3.0	nd			Increase (ELISA): sRANKL Increase (WB)	12…48 12	2.0 2.0
	3D (Coll. gel)	Kang et al. 2013 [44]	2; 48	2.0	Increase (qPCR: GAPDH)	2	2.0			
	3D (PLLA modif.)	Liao et al. 2016 [14]	1 d; 3 d; 7 d; 14 d	5.0; 15.0; 25.0; 35.0	Increase (qPCR: GAPDH)	Day 14	35.0			
	3D (PLGA)	Jianru et al. 2015 [43]	3; 6; 12 (WB: 12)	25.0	Increase (qPCR: GAPDH)	6	25.0	Increase (WB)	12	25.0
		Li et al. 2016 [31]	6; 24; 72	25.0	Increase (qPCR: GAPDH)	6	25.0			
		Li et al. 2016 [27]	6; 24; 72	5.0; 15.0; 25.0	Increase (qPCR: GAPDH)	6	25.0	Decrease (ELISA)	72	25.0
		Li et al. 2011 [33]	6; 24; 72	5; 15; 25; 35	Increase (qPCR: GAPDH) Increase followed by no change (qPCR: GAPDH)	6 6 (increase) 72 (no change)	25…35.0 25 25			
		Yi et al. 2016 [28]	24	25.0	Increase (qPCR: GAPDH)	24	25.0	Increase (WB)	24	25.0

2D: two-dimensional cell culture; 3D (Coll. gel): three-dimensional cell culture, collagen gel; 3D (PLGA): three-dimensional cell culture using PLGA scaffolds; 3D (PLLA modif.): three-dimensional cell culture, hydrophilically modified PLLA scaffolds; qPCR: quantitative polymerase chain reaction (e.g., real-time PCR); sqPCR: semiquantitative polymerase chain reaction, followed by reference gene used; nr: not reported; na: not applicable; ELISA: enzyme-linked immune absorbent assay; WB: Western blot; IF: immunofluorescence; FLM: fluorescence microscopy; EIA: enzyme immunoassay.

**(a):** 

KEGG ID	4060	4668	4510	4620	4370	4062	4380	4010	4064
KEGG name	Cytokine-cytokine receptor interaction	TNF signaling pathway	Focal adhesion	Toll-like receptor signaling pathway	VEGF signaling pathway	Chemokine signaling pathway	Osteoclast differentiation	MAPK signaling pathway	NF-kappa B signaling pathway
False discovery rate	2.62*E*–15	2.06*E*–12	3.90*E*–11	2.04*E*–09	9.47*E*–08	1.33*E*–07	2.29*E*–07	1.42*E*–06	1.86*E*–05
ADRB2									
AKT1		X	X	X	X	X	X	X	
ALPL									
BGLAP									
CBS									
CCL2	X	X				X			
CCL3	X			X		X			
CCL5	X	X		X		X			
CCND1			X						
CCR5	X					X			
CDH11									
COL1A1			X						
COL3A1			X						
COL5A1			X						
CSF1	X	X					X		
CTNNB1			X						
CTSB									
CTSL									
CXCL8 (= IL8)	X			X		X			X
FGF2								X	
GJA1									
GSK3b			X			X			
HMGB1									
HSP90AA1									
HSPA4									
HSPB1					X			X	
IGF1			X						
IL17A	X								
IL1B	X	X		X			X	X	X
IL6	X	X		X					
JAG1		X							
LGALS3BP									
MMP13									
MMP3		X							
PIEZO1									
PLA2G4A					X			X	
POSTN									
PTGS1									
PTGS2		X			X				X
PTK2			X		X	X			
RUNX2									
SPP1			X	X					
TGFB1	X							X	
TGFB3	X						X	X	
TNF	X	X		X			X	X	X
TNFRSF11B	X						X		
TNFSF11	X						X		X
VEGFA	X		X		X				

**(b):** 

KEGG ID	4350	4060	4064	4390	4668	4210	4380	4620	4066
KEGG name	TGF-beta signaling pathway	Cytokine-cytokine receptor interaction	NF-kappa B signaling pathway	Hippo signaling pathway	TNF signaling pathway	Apoptosis	Osteoclast differentiation	Toll-like receptor signaling pathway	HIF-1 signaling pathway
False discovery rate	8.33*E*–23	2.37*E*–21	8.32*E*–11	5.07*E*–09	1.*01E*–08	6.26*E*–08	1.02*E*–05	6.79*E*–05	7.16*E*–05
ACVR1	X	X							
ACVR2A	X	X							
ACVR2B	X	X							
ALPL									
BAX						X			
BCL2			X			X			X
BGLAP									
BMP2	X	X		X					
BMP4	X			X					
BMP6	X			X					
BMP7	X	X		X					
BMPR1A	X	X		X					
BMPR1B	X	X		X					
BMPR2	X	X		X					
Casp3					X	X			
CHRD	X								
CXCR1		X							
FST	X								
GREM1									
IBSP									
IL11		X							
IL11RA									
IL1b		X	X		X	X	X	X	
IL1r1		X	X		X	X			
IL6		X					X	X	X
IL6R		X							X
IL8		X	X				X	X	
MKI67									
MMP1									
MMP13									
MMP14									
MMP2									
MMP3									
NOG	X								
PLAT									
PLAU			X						
PTGS2			X						
RUNX2									
SERPINE1				X					X
SMAD1	X			X					
SP7									
SPP1							X	X	
TIMP1									X
TIMP2									
TIMP3									
TIMP4									
TNF	X	X	X		X	X	X	X	
TNFRSF11B		X							
TNFRSF1A		X	X		X	X			
TNFSF11		X	X						
ZNF354C									

## Abbreviations

2D: Two-dimensional
3D: Three-dimensional
ATP: Adenosine triphosphate
cAMP: Cyclic adenosine monophosphate
ECM: Extracellular matrix
ELISA: Enzyme-linked immunosorbent assay
H_2_S: Hydrogen sulfide
hOBMCs: Human oral bone marrow cells
hOBs: Human osteoblasts
hPDLCs: Human periodontal ligament cells
KEGG: Kyoto encyclopedia of genes and genomes
NO: Nitric oxide
OOF: Optimal orthodontic force
OPG: Osteoprotegerin
OTM: Orthodontic tooth movement
PDL: Periodontal ligament
PGE_2_: Prostaglandin E_2_
PLGA: Polylactic-co-glycolic acid
PLLA: Poly-L-lactide acid
PPI: Protein-protein interaction
PTGS2: Prostaglandin-endoperoxide synthase 2
RANKL: Receptor activator of nuclear factor kappa-*Β* ligand
ROS: Reactive oxygen species
STRING: Search tool for the retrieval of interacting genes/proteins
TNF: Tumor necrosis factor
TNFRSF11B: TNF receptor superfamily member 11b
TNFSF11: TNF superfamily member 11
WAB: Weight approach based.

## Supplementary Materials

Supplementary 1 Search strategy designed for the studies applying the *in vitro* loading model based on a weight approach on cells in 2D or 3D cell culture and lists the excluded studies after full-text reading with reasons.

Supplementary 2 Studies applying the 2D weight approach on human primary cells from the orofacial region, that is, human periodontal ligament cells (hPDLC), human oral bone marrow cells (hOBMC), and human alveolar bone osteoblasts (hOB). For each gene or metabolite force magnitude and force duration, the change in gene expression or substance secretion (increase, decrease, and no change) and the techniques applied are given.

Supplementary 3 Studies applying the 2D weight approach on human and nonhuman cells and cell lines not included in Supplement 2 (i.e., human primary cells from the orofacial region). For each gene or metabolite force magnitude and force duration, the change in gene expression or substance secretion (increase, decrease, and no change) and the techniques applied are given.

Supplementary 4 Studies applying the 3D weight approach on human and nonhuman cells and cell lines. For each gene or metabolite force magnitude and force duration, the change in gene expression or substance secretion (increase, decrease, and no change) and the techniques applied are given.
