# Supplementary material for: In Vitro Weight-Loaded Cell Models for Understanding Mechanodependent Molecular Pathways Involved in Orthodontic Tooth Movement: A Systematic Review
Source: Stem Cells Int. 2018 Jul 31;2018:3208285. doi: 10.1155/2018/3208285 (PMC6091372; doi:10.1155/2018/3208285)
Supplement: Supplementary 1 — Search strategy designed for the studies applying the in vitro loading model based on a weight approach on cells in 2D or 3D cell culture and lists the excluded studies after full-text reading with reasons. [file 3208285.f1.docx]

**Supplement 1**

Janjic et al., *In Vitro* Weight Loaded Cell Models for Understanding Mechano-dependent Molecular Pathways Involved in Orthodontic Tooth Movement: A Systematic Review

**Table 1.** Search strategy designed for the studies applying the *in vitro* loading model based on a weight approach on cells in 2D cell culture.

| **FIELD** |  | **FORCE** |  | **TSSUE/ CELLS** |
| --- | --- | --- | --- | --- |
| orthodont* OR  orthodontic tooth movement OR  orthodontic forces | AND | mechanical stress OR  compress* force OR  continuous* compress* force OR  compressive loading OR  loading OR  compress* OR  mechanical force OR  compressive loading OR  static compressive loading OR  mechanical stress | AND | bone OR  periodontal ligament OR  periodontal ligament cells OR  periodontal ligament fibroblast OR  PDL OR  RAW OR  hPDLCs OR  osteoclast* OR  osteoblast* OR  Saos-2 OR  bone remodelling OR  PBMCs |
| Final look of the prepared entry for the PubMed database:  (orthodont* OR orthodontic tooth movement OR orthodontic forces) AND (mechanical stress OR compress* force OR continuous* compress* force OR compressive loading OR loading OR compress* OR mechanical force OR compressive loading OR static compressive loading OR mechanical stress) AND (bone OR periodontal ligament OR periodontal ligament cells OR periodontal ligament fibroblast OR PDL OR RAW OR hPDLCs OR osteoclast* OR osteoblast* OR Saos-2 OR bone remodelling OR PBMCs) | | | | |

**Table 2.** List of excluded studies after full text reading with reasons – 2D studies.

| **Reason for exclusion (N)** | **Study** |
| --- | --- |
| Another method of force application (31) | Basdra et al. (1997) [1]; Chien et al. (2006) [2]; Chien et al. (2009) [3]; Diercke et al. (2012) [4]; Diercke et al. (2012) [5]; Grimm et al. (2015) [6]; Guo et al. (2015) [7]; Hou et al. (2014) [8]; Imamura et al. (1990) [9]; Ito et al. (2014) [10]; Jacobs et al. (2013) [11]; Konermann et al. (2016) [12]; Korb et al. (2016) [13]; Li et al. (2009) [14]; Li et al. (2013) [15]; Liu et al. (2017) [16]; Liu, et al. (2009) [17]; Maeda et al. (2007) [18]; Maeda et al. (2015) [19]; Morikawa et al. (2016) [20]; Nakao et al. (2007) [21]; Sen et al. (2015) [22]; Shu et al. (2017) [23]; Wang et al. (2015) [24]; Wolf et al. (2016) [25]; Wu et al. (2015) [26]; Xu et al. (2014) [27]; Xu et al. (2015) [28]; Yang et al. (2010) [29]; Zhang et al. (2013) [30]; Zhang et al. (2016) [31] |
| Not in English (3) | Huang et al. (2006) [32]; Jiang et al. (2006) [33]; Xu et al. (2008) [34] |
| Review article (2) | Takano-Yamamoto et al. (2017) [35]; Yamaguchi et al. (2005) [36] |
| Other body part (1) | Ichimiya et al. (2007) [37] |
| Missing full text (1) | Ikeda et al. (2016) [38] |
| *In vivo* (7) | Cobo et al. (2016) [39]; Gluhak-Heinrich et al. (2006) [40]; Hayashi et al. (2012) [41]; Madureira et al. (2012) [42]; Nakano et al. (2015) [43]; Wolf et al. (2013) [44]; Xu et al. (2017) [45] |
| 3D (6) | de Araujo et al. (2007) [46]; de Araujo et al. (2014) [47]; Li et al. (2016a) [48]; Li et al. (2016b) [49]; Liao et al. (2016) [50]; Yi et al. (2016) [51] |

**Table 3.** Search strategy designed for studies applying the *in vitro* loading model based on a weight approach on 3D cell culture.

| **FIELD** |  | **FORCE** |  | **TISSUE/CELLS** |  | **3D MODEL** |
| --- | --- | --- | --- | --- | --- | --- |
| orthodontic force OR  periodont* OR  orthodontic tooth movement OR  tooth movement OR  OTM OR  orthodont* OR  orthodontic force | AND | mechanical stress OR  mechan* stress OR  compressive force OR  static compressive force OR  mechanical loading OR  mechanical stress OR  static compressive force OR  static compress* OR  static force OR  loading OR  compress* OR  compressive loading OR  pressure OR  continuous compressive force OR  continuous compress* OR  Static Compress* OR  mechanical force OR  compressive stress | AND | periodontal ligament cells OR  periodont* OR  periodontal ligament OR  PDL OR  PDL cells OR  periodontal ligament fibroblasts OR  periodontal ligament cells OR  osteoblast* OR  osteoclast* OR  alveolar bone OR  bone resorption OR  PDL tissue OR  human gingival fibroblasts OR  periodontal tissue | AND | three-dimensional culture system OR  collagen OR  collagen gel* OR  three-dimensional model OR  3D OR  3D loading model OR  3-D model OR  in vitro model OR  3-D in vitro model OR  Gels OR  3-D culturing OR  3D culturing OR  poly lactic-co-glycolic acid scaffolds OR  PLGA scaffolds OR  Scaffolds OR  PLGA OR  PDL tissue model OR  Three-Dimensional Cultured OR  three-dimensional gels OR  periodontal ligament tissue model OR  tissue model OR  *in vitro* tissue model* OR  porous poly scaffold OR  periodontal ligament like tissue model |
| Final look of the prepared entry for the PubMed database:  (orthodontic force OR periodont* OR orthodontic tooth movement OR tooth movement OR OTM OR orthodont* OR orthodontic force) AND (mechanical stress OR mechan* stress OR compressive force OR static compressive force OR mechanical loading OR mechanical stress OR static compressive force OR static compress* OR static force OR loading OR compress* OR compressive loading OR pressure OR continuous compressive force OR continuous compress* OR Static Compress* OR mechanical force OR compressive stress) AND (periodontal ligament cells OR periodont* OR periodontal ligament OR PDL OR PDL cells OR periodontal ligament fibroblasts OR periodontal ligament cells OR osteoblast* OR osteoclast* OR alveolar bone OR bone resorption OR PDL tissue OR human gingival fibroblasts OR periodontal tissue) AND (three-dimensional culture system OR collagen OR collagen gel* OR three-dimensional model OR 3D OR 3D loading model OR 3-D model OR in vitro model OR 3-D in vitro model OR Gels OR 3-D culturing OR 3D culturing OR poly lactic-co-glycolic acid scaffolds OR PLGA scaffolds OR Scaffolds OR PLGA OR PDL tissue model OR Three-Dimensional Cultured OR three-dimensional gels OR periodontal ligament tissue model OR tissue model OR in vitro tissue model* OR porous poly scaffold OR periodontal ligament like tissue model) | | | | | | |

**Table 4.** List of excluded studies after full text reading with reasons – 3D studies.

| **Reason for exclusion (N)** | **Study** |
| --- | --- |
| Another method of force application (17) | Berendsen et al. (2009) [52]; Chang et al. (2008) [53]; Chang et al. (2015) [54]; Deschner et al. (2012) [55]; Diercke et al. (2011) [56]; Gharibi et al. (2013) [57]; Guo et al. (2015) [7]; Hou et al. (2014) [8]; Huang et al. (2009) [58]; Jacobs et al. (2013) [11]; Oortgiesen et al. (2012) [59]; Saminathan et al. (2013) [60]; Saminathan et al. (2015) [61]; Wolf et al. (2016) [25]; Wu et al. (2015) [26]; Xu et al. (2017) [62]; Yang et al. (2010) [29]; Zhang et al. (2013) [30]; Zhao et al. (2008) [63] |
| Not in English (2) | An et al. (2009) [64]; Huang et al. (2006) [32] |
| Organ explant (1) | Duncan et al. (1984) [65] |
| Not related to OTM (1) | Tabeian et al. (2017) [66] |
| Infinite element method (1) | Xin et al. (2002) [67] |
| Review article (1) | Wang et al. (2016) [68] |
| *In vivo* (3) | Gluhak-Heinrich et al. (2006) [40]; Moura et al. (2014) [69]; Zhao et al. (2008) [70] |
| Missing full text (1) | Zhang et al. (2016) [71] |
| No force application (1) | Cobo et al. (2016) [39] |
| 2D (5) | Chen et al. (2015) [72]; Feng et al. (2017) [73]; Liu et al. (2017) [74]; Tripuwabhrut et al. (2013) [75]; Wolf et al. (2014) [76] |

**References**

1. E. K. Basdra, "Biological reactions to orthodontic tooth movement," *Journal of Orofacial Orthopedics,* vol. 58, no. 1, pp. 2-15, 1997.

2. C. H. Chien, S. Otsuki, S. A. Chowdhury et al., "Enhancement of cytotoxic activity of sodium fluoride against human periodontal ligament fibroblasts by water pressure," *In Vivo,* vol. 20, no. 6b, pp. 849-56, 2006.

3. C. H. Chien, H. Sakagami, M. Kouhara et al., "Effect of simulated orthodontic forces on fluoride-induced cytotoxicity in MC3T3-E1 osteoblast-like cells," *In Vivo,* vol. 23, no. 2, pp. 259-65, 2009.

4. K. Diercke, A. Kohl, C. J. Lux et al., "IL-1β and compressive forces lead to a significant induction of RANKL-expression in primary human cementoblasts," *Journal of Orofacial Orthopedics,* vol. 73, no. 5, pp. 397-412, 2012.

5. K. Diercke, A. Konig, A. Kohl et al., "Human primary cementoblasts respond to combined IL-1β stimulation and compression with an impaired BSP and CEMP-1 expression," *European Journal of Cell Biology,* vol. 91, no. 5, pp. 402-12, 2012.

6. S. Grimm, C. Walter, A. Pabst et al., "Effect of compressive loading and incubation with clodronate on the RANKL/OPG system of human osteoblasts," *Journal of Orofacial Orthopedics,* vol. 76, no. 6, pp. 531-42, 2015.

7. T. Guo, L. Zhang, A. Konermann et al., "Manganese superoxide dismutase is required to maintain osteoclast differentiation and function under static force," *Scientific Reports,* vol. 5, pp. 8016, 2015.

8. J. Hou, Y. Chen, X. Meng et al., "Compressive force regulates ephrinB2 and EphB4 in osteoblasts and osteoclasts contributing to alveolar bone resorption during experimental tooth movement," *Korean Journal of Orthodontics,* vol. 44, no. 6, pp. 320-9, 2014.

9. K. Imamura, H. Ozawa, T. Hiraide et al., "Continuously applied compressive pressure induces bone resorption by a mechanism involving prostaglandin E_2_ synthesis," *Journal of Cellular Physiology,* vol. 144, no. 2, pp. 222-8, 1990.

10. M. Ito, T. Arakawa, M. Okayama et al., "Gravity loading induces adenosine triphosphate release and phosphorylation of extracellular signal-regulated kinases in human periodontal ligament cells," *J Investig Clin Dent,* vol. 5, no. 4, pp. 266-74, 2014.

11. C. Jacobs, S. Grimm, T. Ziebart et al., "Osteogenic differentiation of periodontal fibroblasts is dependent on the strength of mechanical strain," *Archives of Oral Biology,* vol. 58, no. 7, pp. 896-904, 2013.

12. A. Konermann, A. Kantarci, S. Wilbert et al., "Verification of γ-Amino-Butyric Acid (GABA) signaling system components in periodontal ligament cells in vivo and in vitro," *Cellular and Molecular Neurobiology,* vol. 36, no. 8, pp. 1353-1363, 2016.

13. K. Korb, E. Katsikogianni, S. Zingler et al., "Inhibition of AXUD1 attenuates compression-dependent apoptosis of cementoblasts," *Clinical Oral Investigations,* vol. 20, no. 9, pp. 2333-2341, 2016.

14. J. Li, L. Jiang, G. Liao et al., "Centrifugal forces within usually-used magnitude elicited a transitory and reversible change in proliferation and gene expression of osteoblastic cells UMR-106," *Molecular Biology Reports,* vol. 36, no. 2, pp. 299-305, 2009.

15. F. F. Li, F. L. Chen, H. Wang et al., "Proteomics based detection of differentially expressed proteins in human osteoblasts subjected to mechanical stress," *Biochemistry and Cell Biology,* vol. 91, no. 2, pp. 109-15, 2013.

16. J. Liu, Q. Li, S. Liu et al., "Periodontal ligament stem cells in the periodontitis microenvironment are sensitive to static mechanical strain," *Stem Cells Int,* vol. 2017, pp. 1380851, 2017.

17. J. Liu, Z. Zhao, J. Li et al., "Hydrostatic pressures promote initial osteodifferentiation with ERK1/2 not p38 MAPK signaling involved," *Journal of Cellular Biochemistry,* vol. 107, no. 2, pp. 224-32, 2009.

18. A. Maeda, K. Soejima, K. Bandow et al., "Force-induced IL-8 from periodontal ligament cells requires IL-1β," *Journal of Dental Research,* vol. 86, no. 7, pp. 629-34, 2007.

19. A. Maeda, K. Bandow, J. Kusuyama et al., "Induction of CXCL2 and CCL2 by pressure force requires IL-1β-MyD88 axis in osteoblasts," *Bone,* vol. 74, pp. 76-82, 2015.

20. T. Morikawa, K. Matsuzaka, K. Nakajima et al., "Dental pulp cells promote the expression of receptor activator of nuclear factor-κB ligand, prostaglandin E_2_ and substance P in mechanically stressed periodontal ligament cells," *Archives of Oral Biology,* vol. 70, pp. 158-164, 2016.

21. K. Nakao, T. Goto, K. K. Gunjigake et al., "Intermittent force induces high RANKL expression in human periodontal ligament cells," *Journal of Dental Research,* vol. 86, no. 7, pp. 623-8, 2007.

22. S. Sen, K. Diercke, S. Zingler et al., "Compression induces Ephrin-A2 in PDL fibroblasts via c-fos," *Journal of Dental Research,* vol. 94, no. 3, pp. 464-72, 2015.

23. R. Shu, D. Bai, T. Sheu et al., "Sclerostin promotes bone remodeling in the process of tooth movement," *PLoS One,* vol. 12, no. 1, pp. e0167312, 2017.

24. H. Wang, R. Wang, Z. Wang et al., "ClC-3 chloride channel functions as a mechanically sensitive channel in osteoblasts," *Biochemistry and Cell Biology,* vol. 93, no. 6, pp. 558-65, 2015.

25. M. Wolf, S. Lossdörfer, P. Römer et al., "Short-term heat pre-treatment modulates the release of HMGB1 and pro-inflammatory cytokines in hPDL cells following mechanical loading and affects monocyte behavior," *Clinical Oral Investigations,* vol. 20, no. 5, pp. 923-31, 2016.

26. J. Wu, M. Song, T. Li et al., "The Rho-mDia1 signaling pathway is required for cyclic strain-induced cytoskeletal rearrangement of human periodontal ligament cells," *Experimental Cell Research,* vol. 337, no. 1, pp. 28-36, 2015.

27. H. Xu, X. Han, Y. Meng et al., "Favorable effect of myofibroblasts on collagen synthesis and osteocalcin production in the periodontal ligament," *American Journal of Orthodontics and Dentofacial Orthopedics,* vol. 145, no. 4, pp. 469-79, 2014.

28. H. Xu, D. Bai, L. B. Ruest et al., "Expression analysis of a-smooth muscle actin and tenascin-C in the periodontal ligament under orthodontic loading or *in vitro* culture," *Int J Oral Sci,* vol. 7, no. 4, pp. 232-41, 2015.

29. Y. Yang, Y. Yang, X. Li et al., "Functional analysis of core binding factor a1 and its relationship with related genes expressed by human periodontal ligament cells exposed to mechanical stress," *European Journal of Orthodontics,* vol. 32, no. 6, pp. 698-705, 2010.

30. P. Zhang, Y. Wu, Q. Dai et al., "p38-MAPK signaling pathway is not involved in osteogenic differentiation during early response of mesenchymal stem cells to continuous mechanical strain," *Molecular and Cellular Biochemistry,* vol. 378, no. 1-2, pp. 19-28, 2013.

31. L. Zhang, W. Liu, J. Zhao et al., "Mechanical stress regulates osteogenic differentiation and RANKL/OPG ratio in periodontal ligament stem cells by the Wnt/β-catenin pathway," *Biochimica et Biophysica Acta,* vol. 1860, no. 10, pp. 2211-9, 2016.

32. S. G. Huang, J. X. Zhang, P. Y. Xiong et al., "[Effect of continuously compressive pressure on the expression of RANKL mRNA in human periodontal ligament cells in vitro]," *Zhong Nan Da Xue Xue Bao Yi Xue Ban,* vol. 31, no. 4, pp. 518-22, 2006.

33. L. Y. Jiang, Z. H. Zhao and J. Wang, "[Effects of mechanical tensile stress on the expression of ICAM-1 mRNA in osteoblasts differentiated from rBMSCs]," *Sichuan Da Xue Xue Bao Yi Xue Ban,* vol. 37, no. 3, pp. 438-41, 2006.

34. H. Y. Xu, H. Zhou and G. Z. Rao, "[The effect of continuously compressive press on the shape of cells and expressions of MMP-9,TRAP in human osteoclasts]," *Shanghai Kou Qiang Yi Xue,* vol. 17, no. 3, pp. 285-8, 2008.

35. T. Takano-Yamamoto, T. Fukunaga and N. Takeshita, "Gene expression analysis of CCN protein in bone under mechanical stress," *Methods in Molecular Biology,* vol. 1489, pp. 283-308, 2017.

36. M. Yamaguchi and K. Kasai, "Inflammation in periodontal tissues in response to mechanical forces," *Archivum Immunologiae et Therapiae Experimentalis,* vol. 53, no. 5, pp. 388-98, 2005.

37. H. Ichimiya, T. Takahashi, W. Ariyoshi et al., "Compressive mechanical stress promotes osteoclast formation through RANKL expression on synovial cells," *Oral Surgery, Oral Medicine, Oral Pathology, Oral Radiology and Endodontics,* vol. 103, no. 3, pp. 334-41, 2007.

38. M. Ikeda, Y. Yoshimura, T. Kikuiri et al., "Release from optimal compressive force suppresses osteoclast differentiation," *Mol Med Rep,* vol. 14, no. 5, pp. 4699-4705, 2016.

39. T. Cobo, C. G. Viloria, L. Solares et al., "Role of periostin in adhesion and migration of bone remodeling cells," *PLoS One,* vol. 11, no. 1, pp. e0147837, 2016.

40. J. Gluhak-Heinrich, S. Gu, D. Pavlin et al., "Mechanical loading stimulates expression of connexin 43 in alveolar bone cells in the tooth movement model," *Cell Commun Adhes,* vol. 13, no. 1-2, pp. 115-25, 2006.

41. N. Hayashi, M. Yamaguchi, R. Nakajima et al., "T-helper 17 cells mediate the osteo/odontoclastogenesis induced by excessive orthodontic forces," *Oral Diseases,* vol. 18, no. 4, pp. 375-88, 2012.

42. D. F. Madureira, A. Taddei Sde, M. H. Abreu et al., "Kinetics of interleukin-6 and chemokine ligands 2 and 3 expression of periodontal tissues during orthodontic tooth movement," *American Journal of Orthodontics and Dentofacial Orthopedics,* vol. 142, no. 4, pp. 494-500, 2012.

43. Y. Nakano, M. Yamaguchi, M. Shimizu et al., "Interleukin-17 is involved in orthodontically induced inflammatory root resorption in dental pulp cells," *American Journal of Orthodontics and Dentofacial Orthopedics,* vol. 148, no. 2, pp. 302-9, 2015.

44. M. Wolf, S. Lossdörfer, N. Abuduwali et al., "Potential role of high mobility group box protein 1 and intermittent PTH (1-34) in periodontal tissue repair following orthodontic tooth movement in rats," *Clinical Oral Investigations,* vol. 17, no. 3, pp. 989-97, 2013.

45. H. Xu, Y. He, J. Q. Feng et al., "Wnt3α and transforming growth factor-β induce myofibroblast differentiation from periodontal ligament cells via different pathways," *Experimental Cell Research,* vol. 353, no. 2, pp. 55-62, 2017.

46. R. M. Santos de Araujo, Y. Oba and K. Moriyama, "Role of regulator of G-protein signaling 2 (RGS2) in periodontal ligament cells under mechanical stress," *Cell Biochemistry and Function,* vol. 25, no. 6, pp. 753-8, 2007.

47. R. M. Santos de Araujo, Y. Oba, S. Kuroda et al., "RhoE regulates actin cytoskeleton organization in human periodontal ligament cells under mechanical stress," *Archives of Oral Biology,* vol. 59, no. 2, pp. 187-92, 2014.

48. M. Li, J. Yi, Y. Yang et al., "Investigation of optimal orthodontic force at the cellular level through three-dimensionally cultured periodontal ligament cells," *European Journal of Orthodontics,* vol. 38, no. 4, pp. 366-72, 2016.

49. M. L. Li, J. Yi, Y. Yang et al., "Compression and hypoxia play independent roles while having combinative effects in the osteoclastogenesis induced by periodontal ligament cells," *Angle Orthodontist,* vol. 86, no. 1, pp. 66-73, 2016.

50. W. Liao, M. Okada, K. Inami et al., "Cell survival and gene expression under compressive stress in a three-dimensional in vitro human periodontal ligament-like tissue model," *Cytotechnology,* vol. 68, no. 2, pp. 249-60, 2016.

51. J. Yi, B. Yan, M. Li et al., "Caffeine may enhance orthodontic tooth movement through increasing osteoclastogenesis induced by periodontal ligament cells under compression," *Archives of Oral Biology,* vol. 64, pp. 51-60, 2016.

52. A. D. Berendsen, T. H. Smit, X. F. Walboomers et al., "Three-dimensional loading model for periodontal ligament regeneration in vitro," *Tissue Eng Part C Methods,* vol. 15, no. 4, pp. 561-70, 2009.

53. H. H. Chang, C. B. Wu, Y. J. Chen et al., "MMP-3 response to compressive forces in vitro and in vivo," *Journal of Dental Research,* vol. 87, no. 7, pp. 692-6, 2008.

54. M. Chang, H. Lin, M. Luo et al., "Integrated miRNA and mRNA expression profiling of tension force-induced bone formation in periodontal ligament cells," *In Vitro Cellular and Developmental Biology. Animal,* vol. 51, no. 8, pp. 797-807, 2015.

55. B. Deschner, B. Rath, A. Jager et al., "Gene analysis of signal transduction factors and transcription factors in periodontal ligament cells following application of dynamic strain," *Journal of Orofacial Orthopedics,* vol. 73, no. 6, pp. 486-95, 497, 2012.

56. K. Diercke, S. Sen, A. Kohl et al., "Compression-dependent up-regulation of ephrin-A2 in PDL fibroblasts attenuates osteogenesis," *Journal of Dental Research,* vol. 90, no. 9, pp. 1108-15, 2011.

57. B. Gharibi, G. Cama, M. Capurro et al., "Gene expression responses to mechanical stimulation of mesenchymal stem cells seeded on calcium phosphate cement," *Tissue Engineering Part A,* vol. 19, no. 21-22, pp. 2426-38, 2013.

58. L. Huang, Y. Meng, A. Ren et al., "Response of cementoblast-like cells to mechanical tensile or compressive stress at physiological levels in vitro," *Molecular Biology Reports,* vol. 36, no. 7, pp. 1741-8, 2009.

59. D. A. Oortgiesen, N. Yu, A. L. Bronckers et al., "A three-dimensional cell culture model to study the mechano-biological behavior in periodontal ligament regeneration," *Tissue Eng Part C Methods,* vol. 18, no. 2, pp. 81-9, 2012.

60. A. Saminathan, K. J. Vinoth, H. H. Low et al., "Engineering three-dimensional constructs of the periodontal ligament in hyaluronan-gelatin hydrogel films and a mechanically active environment," *Journal of Periodontal Research,* vol. 48, no. 6, pp. 790-801, 2013.

61. A. Saminathan, G. Sriram, J. K. Vinoth et al., "Engineering the periodontal ligament in hyaluronan-gelatin-type I collagen constructs: upregulation of apoptosis and alterations in gene expression by cyclic compressive strain," *Tissue Engineering Part A,* vol. 21, no. 3-4, pp. 518-29, 2015.

62. H. Y. Xu, E. M. Nie, G. Deng et al., "Periostin is essential for periodontal ligament remodeling during orthodontic treatment," *Mol Med Rep,* vol. 15, no. 4, pp. 1800-1806, 2017.

63. Y. Zhao, C. Wang, S. Li et al., "Expression of Osterix in mechanical stress-induced osteogenic differentiation of periodontal ligament cells in vitro," *European Journal of Oral Sciences,* vol. 116, no. 3, pp. 199-206, 2008.

64. Y. Y. An, H. Zhou, Y. S. Ruan et al., "[Mass chromatographic analysis on different protein expression of human periodontal ligament cell under static pressure]," *Shanghai Kou Qiang Yi Xue,* vol. 18, no. 1, pp. 56-60, 2009.

65. G. W. Duncan, E. H. Yen, E. T. Pritchard et al., "Collagen and prostaglandin synthesis in force-stressed periodontal ligament *in vitro*," *Journal of Dental Research,* vol. 63, no. 5, pp. 665-9, 1984.

66. H. Tabeian, A. D. Bakker, B. F. Betti et al., "Cyclic Tensile Strain Reduces TNF-alpha Induced Expression of MMP-13 by Condylar Temporomandibular Joint Cells," *Journal of Cellular Physiology,* vol. 232, no. 6, pp. 1287-1294, 2017.

67. H. Xin, X. Ma, L. Ying et al., "The application of infinite element method to endodontic endosseous implant stress analysis," *Zhonghua Kou Qiang Yi Xue Za Zhi,* vol. 37, no. 3, pp. 183-6, 2002.

68. T. Wang, G. Li, J. Chen et al., "Three-dimensional stress In vitro promotes the proliferation and differentiation of periodontal ligament stem cells implanted by bioactive glass," *Cell Mol Biol (Noisy-le-grand),* vol. 62, no. 10, pp. 62-7, 2016.

69. A. P. Moura, S. R. Taddei, C. M. Queiroz-Junior et al., "The relevance of leukotrienes for bone resorption induced by mechanical loading," *Bone,* vol. 69, pp. 133-8, 2014.

70. Z. Zhao, Y. Fan, D. Bai et al., "The adaptive response of periodontal ligament to orthodontic force loading - a combined biomechanical and biological study," *Clin Biomech (Bristol, Avon),* vol. 23 Suppl 1, pp. S59-66, 2008.

71. X. Zhang, W. G. Guo, H. Cui et al., "In vitro and in vivo enhancement of osteogenic capacity in a synthetic BMP-2 derived peptide-coated mineralized collagen composite," *J Tissue Eng Regen Med,* vol. 10, no. 2, pp. 99-107, 2016.

72. Y. Chen, A. Mohammed, M. Oubaidin et al., "Cyclic stretch and compression forces alter microRNA-29 expression of human periodontal ligament cells," *Gene,* vol. 566, no. 1, pp. 13-7, 2015.

73. L. Feng, Y. Zhang, X. Kou et al., "Cadherin-11 modulates cell morphology and collagen synthesis in periodontal ligament cells under mechanical stress," *Angle Orthodontist,* vol. 87, no. 2, pp. 193-199, 2017.

74. F. Liu, F. Wen, D. He et al., "Force-induced H_2_S by PDLSCs modifies osteoclastic activity during tooth movement," *Journal of Dental Research,* vol. 96, no. 6, pp. 694-702, 2017.

75. P. Tripuwabhrut, M. Mustafa, C. G. Gjerde et al., "Effect of compressive force on human osteoblast-like cells and bone remodelling: an *in vitro* study," *Archives of Oral Biology,* vol. 58, no. 7, pp. 826-36, 2013.

76. M. Wolf, S. Lossdörfer, K. Küpper et al., "Regulation of high mobility group box protein 1 expression following mechanical loading by orthodontic forces *in vitro* and *in vivo*," *European Journal of Orthodontics,* vol. 36, no. 6, pp. 624-31, 2014.
