# Supplementary material for: In Vitro Weight-Loaded Cell Models for Understanding Mechanodependent Molecular Pathways Involved in Orthodontic Tooth Movement: A Systematic Review
Source: Stem Cells Int. 2018 Jul 31;2018:3208285. doi: 10.1155/2018/3208285 (PMC6091372; doi:10.1155/2018/3208285)
Supplement: Supplementary 2 — Studies applying the 2D weight approach on human primary cells from the orofacial region, that is, human periodontal ligament cells (hPDLC), human oral bone marrow cells (hOBMC), and human alveolar bone osteoblasts (hOB). For each gene or metabolite force magnitude and force duration, the change in gene expression or substance secretion (increase, decrease, and no change) and the techniques applied are given. [file 3208285.f2.docx]

# Supplement 2. Studies applying the 2D weight approach on human primary cells from the orofacial region, i.e. human periodontal ligament cells (hPDLC), human oral bone marrow cells (hOBMC), and human alveolar bone osteoblasts (hOB). For each gene or metabolite force magnitude and force duration, the change in gene expression or substance secretion (increase, decrease, no change), and the techniques for analysis applied are given.

| **Reference** | **Cell type^a^** | **Gene/ metabolite symbol** | **Examined force applied** | | **Gene expression ^b, c^**  **(Increase/ decrease/ no change)** | **Substance secretion ^c,d^**  **(Increase/ decrease/ no change)** |
| --- | --- | --- | --- | --- | --- | --- |
|  |  |  | **Magnitude [g/cm^2^]** | **Duration [h]** |  |  |
| Asano et al. 2011 [1] | hPDLC (exp) | *CXCL8* | 1.0; 2.0; 3.0; 4.0 | 0; 3; 6; 9; 12; 24 | Increase (qPCR: GAPDH) | Increase (ELISA) |
|  |  | *CCL2* | 1.0; 2.0; 3.0; 4.0 | 0; 3; 6; 9; 12; 24 | Increase (qPCR: GAPDH) | Increase (ELISA) |
| Benjakul et al. in press [2] | hPDLC (exp?) | PGE_2_ | 1.5 | 48 | n. a. | Increase (ELISA) |
|  |  | *TNFSF11* | 1.5 | 48 | Increase (qPCR: GAPDH) | Increase (ELISA) |
|  |  | *TNFRSF11B* | 1.5 | 48 | No change (qPCR: GAPDH) | No change (ELISA) |
|  |  | *RUNX2* | 1.5 | 48 | Decrease (qPCR: GAPDH) | n. r. |
| Cao et al. 2014 [3] | hPDLC (dig) | *ADRB2* | 1.5  0.5; 1.0; 1.5; 2.0 | 0; 2; 4; 6; 8; 12  6 | n. r | Increase (WB) |
|  | hOBMC | *ADRB2* | 1.5  0.5; 1.0; 1.5; 2.0 | 0; 2; 4; 6; 8; 12  6 | n. r | No change (WB) |
| Chae et al. 2011 [4] | hPDLC ($$) | ROS | 3.0 | 4 | n. a. | Increase (FLM) |
|  |  | *IL1B* | 3.0 | 24 | Increase (qPCR: GAPDH) | Increase (ELISA) |
|  |  | *CXCL8* | 3.0 | 24 | Increase (qPCR: GAPDH) | Increase (ELISA) |
|  |  | *IL6* | 3.0 | 24 | Increase (qPCR: GAPDH) | Increase (ELISA) |
|  |  | *TNF* | 3.0 | 24 | Increase (qPCR: GAPDH) | Increase (ELISA) |
| Chen et al. 2015 [5] | hPDLC (exp) | *COL1A1* | 2.0 | 24 | Decrease (qPCR: ACTB) | n. r. |
|  |  | *COL3A1* | 2.0 | 24 | Decrease (qPCR: ACTB) | n. r. |
|  |  | *COL5A1* | 2.0 | 24 | No change (qPCR: ACTB) | n. r. |
|  |  | microRNAs | 2.0 | 24 | Increase (qPCR: U6snRNA) | n. r. |
| Feng et al. 2017 [6] | hPDLC (dig) | *CDH11* | 0.5; 1.0; 1.5; 2.0  1.0 | 24  4; 8; 12; 24 | Decrease (qPCR: GAPDH) | Decrease (WB) |
|  |  | *COL1A1* | 0.5; 1.0; 1.5; 2.0  1.0 | 24  4; 8; 12; 24 | Decrease (qPCR: GAPDH) | Decrease (WB) |
|  |  | *CTNNB1* | 0.5; 1.0; 1.5; 2.0  1.0 | 24  4; 8; 12; 24 | n. r. | Decrease (WB) |
| Feng et al. 2016 [7] | hPDLC (dig) | *COL1A1* | 1.0 | 24 | Decrease (qPCR: GAPDH) | n. r. |
|  |  | *TGFB1* | 1.0 | 24 | Decrease (qPCR: GAPDH) | n. r. |
|  |  | *TGFB3* | 1.0 | 24 | Decrease (qPCR: GAPDH) | n. r. |
|  |  | TGF-β (*antibody specificity not identifiable) | 1.0 | 24 | n. a. | Decrease (WB) |
| He et al. 2015 [8] | hPDLC (dig) | Effect on macro­phages in co-culture | 1 | 24 | n. r. | n. r. |
| Jin et al. 2015 [9] | hPDLC (dig) | *PTGS2* | 2.0 | 0.5; 3; 6; 12 | Increase (qPCR: GAPDH) | n. r. |
|  |  | NFKB (*antibody specificity not identifiable) | 2.0 | 3 | n. r. | Increased nuclear translocation (WB) |
|  |  | *TNFRSF11B* | 2.0 | 0.5; 3; 6; 12 | No change (qPCR: GAPDH) | n. r. |
|  |  | PGE_2_ | 2.0 | 12 | n. a. | Increase (ELISA) |
|  |  | *PIEZO1* | 2.0 | 0.5; 3; 6; 12  WB: 3 | Increase (qPCR: GAPDH) | Increase (WB) |
|  |  | *TNFSF11* | 2.0 | 0.5; 3; 6; 12 | Increase (qPCR: GAPDH) | n. r. |
| Kang et al. 2013 [10] | hPDLC (dig) | *IL1B* | 2.0 | 2; 48 | No change (qPCR: GAPDH) | n. r. |
|  |  | *TNF* | 2.0 | 2; 48 | No change (qPCR: GAPDH) | n. r. |
|  |  | *TNFSF11* | 2.0 | 2; 48 | Increase (qPCR: GAPDH) | n. r. |
|  |  | *MMP3* | 2.0 | 2; 48 | Decrease (qPCR: GAPDH) | n. r. |
|  |  | *MMP13* | 2.0 | 2; 48 | Increase (qPCR: GAPDH) | n. r. |
| Kang et al. 2010 [11] | hPDLC (?) | *PTGS2* | 2.0 | 0.5; 2; 6; 24; 48 | Increase (qPCR: GAPDH) | n. r. |
|  |  | *PTK2* | 2.0 | 0.5; 2; 6; 24; 48 | n. r. | p-FAK: Increase (WB) overall FAK: no change (WB) |
|  |  | PGE_2_ | 2.0 | 0.5; 2; 6; 24; 48 | n. a. | Increase (ELISA) |
| Kanjanamekanant et al. 2013 [12] | hPDLC (?) | *IL1B* | 1.0; 1.5; 2.0; 2.5 | 1, 3, 5 | Increase (sqPCR: GAPDH) | Increase (ELISA) |
| Kanjanamekanant et al. 2014 [13] | hPDLC (?) | *IL1B* | 0; 0.5; 1.0; 1.5; 2.0; 2.5 | 3 | n. r. | ELISA |
|  |  | ATP | 2.0 | 3 | n. a. | ELISA |
| Kanzaki et al. 2002 [14] | hPDLC (exp) | *PTGS1* | 0.5; 1.0; 2.0; 3.0; 4.0^+^ | 0.5; 1.5; 6; 24; 48 | No change (sqPCR: ACTNB) | n. r. |
|  |  | *PTGS2* | 0.5; 1.0; 2.0; 3.0; 4.0^+^ | 0.5; 1.5; 6; 24; 48 | Increase (sqPCR: ACTNB) | n. r. |
|  |  | *TNFRSF11B* | 0.5; 1.0; 2.0; 3.0; 4.0^+^ | 0.5; 1.5; 6; 24; 48 | No change (sqPCR: ACTNB) | n. r. |
|  |  | PGE_2_ | 2.0 | 0.5; 1.5; 6; 24; 48,60 | n. a. | Increase (ELISA) |
|  |  | *TNFSF11* | 0.5; 1.0; 2.0; 3.0; 4.0  WB: 2.0 | 0.5; 1.5; 6; 24; 48  WB: 48, 96 | Increase (sqPCR: ACTNB) | Increase (WB): 40-kDa +  55-kDa |
| Kikuta et al. 2015 [15] | hPDLC (exp) | *IL6* | 4.0 | 1; 3; 6; 9; 12; 24 ELISA: 1; 3; 6; 9; 12; 24; 48 | Increase (qPCR: GAPDH) | Increase (ELISA) |
|  |  | *JAG1* | 4.0 | 1; 3; 6; 9; 12; 24 ( | Increase (qPCR: GAPDH) | Increase (ELISA) |
|  |  | *TNFSF11* | 4.0 | 1; 3; 6; 9; 12; 24 ELISA: 1; 3; 6; 9; 12; 24; 48 | Increase (qPCR: GAPDH) | Increase (ELISA) |
| Kim et al. 2013 [16] | hPDLC (dig) | *PTK2* | 2.0 | 0.5; 2; 6; 24; 48 | n. r. | p-FAK/FAK-ratio: Increase (WB) |
|  |  | *CSF1* | 2.0 | 0.5; 2; 6; 24; 48 | Increase (qPCR: GAPDH) | Increase (ELISA) |
|  |  | *TNFRSF11B* | 2.0 | 0.5; 2; 6; 24; 48 | Transitory downregulated. (qPCR: GAPDH) | Transitory down­regulation (ELISA) |
|  |  | *TNFSF11* | 2.0 | 0.5; 2; 6; 24; 48 | Increase (qPCR: GAPDH) | Increase (ELISA) |
|  |  | *TNF* | 2.0 | 0.5; 2; 6; 24; 48 | Increase (qPCR: GAPDH) | Increase (ELISA) |
| Kirschneck et al. 2015 [17] | hPDLC (exp) | *PTGS2* | 2.0 | 24 | Increase (qPCR: POL2RA) | n. r. |
|  |  | *IL6* | 2.0 | 24 | Increase (qPCR: POL2RA) | Not explicitly stated (ELISA) |
|  |  | *TNFRSF11B* | 2.0 | 24 | No change (qPCR: POL2RA) | n. r. |
|  |  | PGE_2_ | 2.0 | 24 | n. a. | Not explicitly stated (ELISA) |
|  |  | *TNFSF11* | 2.0 | 24 | Increase (qPCR: POL2RA) | n. r. |
| Kunii et al. 2013 [18] | hPDLC (exp) | *IL6* | 1.0; 2.0; 3.0; 4.0 | 3; 6; 9; 12; 24  ELISA: 3; 6; 9; 12; 24; 48; 72 | Increase (qPCR: GAPDH) | Increase (ELISA) |
| Lee et al. 2015 [19] | hPDLC (?) | *CCL3* | 2.5 | 2; 4; 8; 24; 48 WB: 24; 48; 72; 96 | Increase (qPCR: ACTNB) | Increase (WB) |
|  |  | *CCL5* | 2.5 | 2; 4; 8; 24; 48 WB: 24; 48; 72; 96 | Increase (qPCR: ACTNB) | Increase (WB) |
|  |  | *CCR5* | 2.5 | 2; 4; 8; 24; 48 WB: 24; 48; 72; 96 | Increase (qPCR: ACTNB) | Increase (WB) |
|  |  | *ALPL* | 2.5 | 24 | Increase (qPCR: ACTNB) | n. r. |
|  |  | *RUNX2* | 2.5 | 24 | No change (qPCR: ACTNB) | n. r. |
|  |  | *BGLAP* | 2.5 | 24 | No change (qPCR: ACTNB) | n. r. |
|  |  | *TNFSF11* | 2.5 | 24 | Increase (qPCR: ACTNB) | n. r. |
|  |  | *TNFRSF11B* | 2.5 | 24 | No change (qPCR: ACTNB) | n. r. |
|  |  | *POSTN* | 2.5 | 24 | Increase (qPCR: ACTNB) | n. r. |
|  |  | *IL12 (*forward and reverse primers are identical. Primer Blast- no results)* | 2.5 | 24 | No change (qPCR: ACTNB) | n. r. |
|  |  | *COL1A1* | 2.5 | 2; 4; 8; 24; 48 | Increase (qPCR: ACTNB) | n. r. |
| Liu et al. 2017 [20] | hPDLC (dig) | *CBS* | 0.5; 1.0; 1.5 | 6; 12; 24 | n. r. | Increase (WB) |
|  |  | H_2_S | 0.5; 1.0; 1.5 | 6; 12; 24 | n. a. | Increase |
|  |  | *CCL2* | 0.5; 1.0; 1.5 | 6; 12; 24 | n. r. | Increase (WB) |
|  |  | *TNFSF11* | 0.5; 1.0; 1.5 | 6; 12; 24 | n. r. | Increase (WB) |
|  |  | *TNFRSF11B* | 0.5; 1.0; 1.5 | 6; 12; 24 | n. r. | Decrease (WB) |
| Liu et al. 2006 [21] | hPDLC (?) | *PTGS2* | 2.0 | 48 | Increase (sqPCR: ACTNB) | n. r. |
|  |  | *IL1B* | 2.0 | 48 | n. r. | No change (ELISA) |
|  |  | PGE_2_ | 2.0 | 48 | n. a. | Increase (ELISA) |
|  |  | *TNFSF11* | 2.0 | 48 | Increase (sqPCR: ACTNB) | n. r. |
|  |  | NO | 2.0 | 48 | n. a. | Increase (HPLC-Griess) |
| Luckprom et al. 2011 [22] | hPDLC (?) | *TNFRSF11B* | 2.5 | 2; 4 | No change (sqPCR: GAPDH) | n. r. |
|  |  | *TNFSF11* | 2.5 | 2; 4 | Increase (sqPCR: GAPDH) | Increase (WB) |
|  |  | ATP | 2.5 | 2; 4 | n. a. | Increase (WB) |
|  |  | *GJA1* | 2.5 | 2; 4 | n. r. (sqPCR: GAPDH) | n. r. |
| Mayahara et al. 2007 [23] | hPDLC (exp) | *PTGS2* | 2 | 3; 6; 12; 24; 48 | Increase (qPCR: GAPDH) | n. r. |
|  |  | PGE_2_ | 2 | 3; 6; 12; 24; 48 | n. a. | Increase (ELISA) |
| Mayahara et al. 2010 [24] | hPDLC (exp) | *PTGS2* | 2.0 | 3; 6; 12; 24; 48 | Increase (qPCR: GAPDH) | n. r. |
|  |  | *PLA2G4A* | 2.0 | 3; 6; 12; 24; 48 | Increase (qPCR: GAPDH) | n. r. |
| Mitsuhashi et al. 2011 [25] | hPDLC (exp) | *HSPB1* | 4.0 | 1; 3; 6; 9; 12; 24 | No change (qPCR: ACTNB) | n. r. |
|  |  | *HSPA4* | 1.0; 2.0; 4.0 | 1; 3; 6; 9; 12; 24 | Increase (qPCR: ACTNB) | Increase (ELISA; WB) |
|  |  | *HSP90AA1* | 4.0 | 1; 3; 6; 9; 12; 24 | Increase (qPCR: ACTNB) | n. r. |
|  |  | *TNFRSF11B* | 4.0 | 1; 3; 6; 9; 12; 24 | No change (qPCR: ACTNB) | n. r. |
|  |  | *TNFSF11* | 4.0 | 1; 3; 6; 9; 12; 24 | Temporary increase (qPCR: ACTNB) | n. r. |
|  |  | *TNF* | 4.0 | 1; 3; 6; 9; 12; 24 | Temporary increase (qPCR: ACTNB) | n. r. |
| Nakajima et al. 2008 [26] | hPDLC (exp) | *FGF2* | 0.5; 1.0; 2.0; 3.0; 4.0 | 1; 3; 6; 9; 12; 24 | Increase (sqPCR: ACTNA) | Increase (ELISA) |
|  |  | *TNFRSF11B* | 0.5; 1.0; 2.0; 3.0; 4.0 | 1; 3; 6; 9; 12; 24 | n. r. | Increase (ELISA) |
|  |  | *TNFSF11* | 0.5; 1.0; 2.0; 3.0; 4.0 | 1; 3; 6; 9; 12; 24 | n. r. | Increase (ELISA) |
| Nishijima et al. 2006 [27] | hPDLC (exp) | *TNFSF11* | 0.5; 1.0; 2.0; 3.0 | 48 | n. r. | Increase (ELISA) |
|  |  | *TNFRSF11B* | 0.5; 1.0; 2.0; 3.0 | 48 | n. r. | Decrease (ELISA) |
| Premaraj et al. 2011 [28] | hPDLC ($$) | *AKT1* | 0.2; 2.2; 5.0 | 6 | n. r. | WB: Increase p-Akt |
|  |  | *GSK3b* | 0.2; 2.2; 5.0 | 6 | n. r. | WB: Increase in p-GSK-3β |
|  |  | *CTNNB1* | 0.2; 2.2; 5.0 | 6 | n. r. | WB: Increase in nuclear dephos-β-catenin |
| Premaraj et al. 2013 [29] | hPDLC ($$) | *AKT1* | 5.0 | 6 | n. r. | Increase in dephos-Akt (WB) |
|  |  | *PTGS2* | 0.2; 2.2; 5.0 | 6 | n. r. | Increase (WB) |
|  |  | *CCND1* | 0.2; 2.2; 5.0 | 6 | n. r. | Increase (WB) |
|  |  | *PTK2* | 0.2; 2.2; 5.0 | 0.5; 1; 3; 6 | n. r. | Increase in p-FAK (WB) |
|  |  | PGE_2_ | 5.0 | 0.5; 1; 3; 6 | n. a. | Increase (ELISA) |
|  |  | *CTNNB1* | 5.0 | 6 | n. r. | Increase in dephos-β-catenin |
|  |  | NO | 5.0 | 0.2; 0.5; 1; 2 | n. a. | Increase (Griess Reagent System) |
| Proff et al. 2014 [30] | hPDLC (exp) | *PTGS2* | 2 | 24 | Increase (qPCR: POLR2A) | Increase (WB) |
|  |  | *IGF1* | 2 | 24 | Increase (qPCR: POLR2A) | n. r. |
|  |  | *IL6* | 2 | 24 | No change (qPCR: POLR2A) | n. r. |
|  |  | *CXCL8* | 2 | 24 | Increase (qPCR: POLR2A) | Decrease (WB, ELISA) |
|  |  | *MMP13* | 2 | 24 | Increase (qPCR: POLR2A) | n. r. |
|  |  | *VEGFA* | 2 | 24 | No change (qPCR: POLR2A) | n. r. |
|  |  | PGE_2_ | 2 | 24 | n. a. | Increase (ELISA) |
| Römer et al. 2013 [31] | hPDLC (exp) | *PTGS2* | 2 | 24 | Increase (qPCR: POLR2A) | n. r. |
|  |  | *TNFRSF11B* | 2 | 24 | No change (qPCR: POLR2A) | n. r. |
|  |  | PGE_2_ | 2 | 24 | n. a. | Increase (ELISA) |
|  |  | *TNFSF11* | 2 | 24 | Increase (qPCR: POLR2A) | n. r. |
| Tripuwabhrut et al. 2013 [32] | hOB | *COL1* | 2.0; 4.0 | 24  ELISA: 24; 72; 7d | Increase (qPCR: GAPDH) | Increase (ELISA) |
|  |  | *TNFSF11* | 2.0; 4.0 | 24; IF: +72 | Increase (qPCR: GAPDH) | IF; not detectable with ELISA |
|  |  | *TNFRSF11B* | 2.0; 4.0 | 24;  ELISA: 24;72 | Decrease (qPCR: GAPDH) | Decrease (ELISA) |
|  |  | PGE_2_ | 2.0; 4.0 | 24 | n. a. | Increase (ELISA) |
|  |  | *SPP1* | 2.0; 4.0 | 24 | No change (qPCR: GAPDH) | n. r. |
|  |  | *BGLAP* | 2.0; 4.0 | 24 | No change (qPCR: GAPDH) | n. r. |
|  |  | *RUNX2* | 2.0; 4.0 | 24 | Decrease (qPCR: GAPDH) | n. r. |
|  |  | *ALPL* | 2.0; 4.0 | 24;  Activity: 24; 72; 7d | Increase (qPCR: GAPDH) | Activity: Increase extracellular  Activity: decrease intracellular |
| Tripuwabhrut et al. 2012 [33] | hOB | *MKI67* | 2.0; 4.0 | 24 | Decrease (qPCR: GAPDH) | n. r. |
|  |  | *BAX* | 2.0; 4.0 | 24 | No change (qPCR: GAPDH) | n. r. |
|  |  | *BCL2* | 2.0; 4.0 | 24 | No change (qPCR: GAPDH) | n. r. |
|  |  | *IL6* | 2.0; 4.0 | 24 | Increase (qPCR: GAPDH) | Decrease (ELISA) |
|  |  | *CXCL8* | 2.0; 4.0 | 24 | Increase (qPCR: GAPDH) | Decrease (ELISA) |
| Wolf et al. 2014 [34] | hPDLC (?) | *HMGB1* | 4.0 | 8 | n. r. | Increase (ELISA) |
| Wolf et al. 2013 [35] | hPDLC (?) | *HMGB1* | 4.0 | 24 | n. r. | Translocation to cytoplasm (IF)  Increase (ELISA) |
| Wongkhantee et al. 2007 [36] | hPDLC (exp) | *PTGS2* | 1.25; 2.5 | 24 | Increase (sqPCR: GAPDH) | n. r. |
|  |  | *SPP1* | 0.5; 0.75; 1.0; 1.25;2.5 | 1; 4; 8; 24; 48 | Increase (sqPCR: GAPDH) | Increase (WB) |
|  |  | *TNFSF11* | 1.25; 2.5 | 24 | Increase (sqPCR: GAPDH) | Increase (WB) |
| Yamada et al. 2013 [37] | hPDLC (exp) | *IL6* | 4.0 | 12 | Increase (qPCR: GAPDH) | Increase (ELISA) |
|  |  | *IL17A* | 4.0 | 12 | n. r. | No change (ELISA) |
|  |  | *TNFRSF11B* | 4.0 | 12 | Decrease (qPCR: GAPDH) | Decrease (ELISA) |
|  |  | *TNFSF11* | 4.0 | 12 | Increase (qPCR: GAPDH) | Increase (ELISA) |
| Yamaguchi et al. 2004 [38] | hPDLC (exp) | *CTSB* | 0.5; 1.0; 2.0; 3.0 | 3; 6; 9; 12; 24 | Increase (sqPCR: GAPDH) | Increase (ELISA) |
|  |  | *CTSL* | 0.5; 1.0; 2.0; 3.0 | 3; 6; 9; 12; 24 | Increase (sqPCR: GAPDH) | Increase (ELISA) |
| Yamaguchi et al. 2006 [39] | hPDLC (exp) | *TNFRSF11B* | 0.5; 1.0; 2.0; 3.0 | 3; 6; 9; 12; 24; 48 | n. r. | Decrease (ELISA) |
|  |  | *TNFSF11* | 0.5; 1.0; 2.0; 3.0 | 3; 6; 9; 12; 24; 48 | n. r. | Increase (ELISA): sRANKL  Increase (WB) |
| Zhang et al. 2017 [40] | hPDLC (dig) | *LGALS3BP* | 0.5; 1.0; 1.5; 2.0; | 24 | n. r. | Increase (ELISA): |

^a^ hPDLC (exp) – hPDLC isolated with explant method; hPDLC (dig) – hPDLC isolated with digestion method, hPDLC (?) – hPDLC, isolation method not given; hPDLC ($$) – hPDLC from commercial sources; hOB – human osteoblasts; hOBMC – human oral bone marrow cells

^b^ qPCR – quantitative PCR (e.g. real time PCR); sqPCR – semi-quantitative PCR; followed by reference gene used

^c^ n. r. – not reported; n. a. – not applicable

^d^ ELISA – Enzyme linked immune absorbent assay; WB – western blot; IF – immunofluorescence; FLM, fluorescence microscopy; HPLC-Griess – High Pressure Liquid Chromatography, Griess detection method; p-FAK/FAK – phosphorylated and non-phosphorylated focal adhesion kinase (FAK); kDa – kilo Dalton; pAkt – phosphorylated protein kinase B; p-GSK-3β – phosphorylated glycogen synthase kinase-3-beta; dephos-β-catenin – dephosphorylated β-catenin; sRANKL – soluble RANKL

References

1. M. Asano, M. Yamaguchi, R. Nakajima et al., "IL-8 and MCP-1 induced by excessive orthodontic force mediates odontoclastogenesis in periodontal tissues," *Oral Diseases,* vol. 17, no. 5, pp. 489-98, 2011.

2. S. Benjakul, S. Jitpukdeebodintra and C. Leethanakul, "Effects of low magnitude high frequency mechanical vibration combined with compressive force on human periodontal ligament cells *in vitro*," *European Journal of Orthodontics,* in press.

3. H. Cao, X. Kou, R. Yang et al., "Force-induced Adrb2 in periodontal ligament cells promotes tooth movement," *Journal of Dental Research,* vol. 93, no. 11, pp. 1163-9, 2014.

4. H. S. Chae, H. J. Park, H. R. Hwang et al., "The effect of antioxidants on the production of pro-inflammatory cytokines and orthodontic tooth movement," *Molecules and Cells,* vol. 32, no. 2, pp. 189-96, 2011.

5. Y. Chen, A. Mohammed, M. Oubaidin et al., "Cyclic stretch and compression forces alter microRNA-29 expression of human periodontal ligament cells," *Gene,* vol. 566, no. 1, pp. 13-7, 2015.

6. L. Feng, Y. Zhang, X. Kou et al., "Cadherin-11 modulates cell morphology and collagen synthesis in periodontal ligament cells under mechanical stress," *Angle Orthodontist,* vol. 87, no. 2, pp. 193-199, 2017.

7. L. Feng, R. Yang, D. Liu et al., "PDL progenitor-mediated PDL recovery contributes to orthodontic relapse," *Journal of Dental Research,* vol. 95, no. 9, pp. 1049-56, 2016.

8. D. He, X. Kou, R. Yang et al., "M1-like macrophage polarization promotes orthodontic tooth movement," *Journal of Dental Research,* vol. 94, no. 9, pp. 1286-94, 2015.

9. Y. Jin, J. Li, Y. Wang et al., "Functional role of mechanosensitive ion channel Piezo1 in human periodontal ligament cells," *Angle Orthodontist,* vol. 85, no. 1, pp. 87-94, 2015.

10. K. L. Kang, S. W. Lee, Y. S. Ahn et al., "Bioinformatic analysis of responsive genes in two-dimension and three-dimension cultured human periodontal ligament cells subjected to compressive stress," *Journal of Periodontal Research,* vol. 48, no. 1, pp. 87-97, 2013.

11. Y. G. Kang, J. H. Nam, K. H. Kim et al., "FAK pathway regulates PGE_2_ production in compressed periodontal ligament cells," *Journal of Dental Research,* vol. 89, no. 12, pp. 1444-9, 2010.

12. K. Kanjanamekanant, P. Luckprom and P. Pavasant, "Mechanical stress-induced interleukin-1beta expression through adenosine triphosphate/P2X7 receptor activation in human periodontal ligament cells," *Journal of Periodontal Research,* vol. 48, no. 2, pp. 169-76, 2013.

13. K. Kanjanamekanant, P. Luckprom and P. Pavasant, "P2X7 receptor-Pannexin1 interaction mediates stress-induced interleukin-1 beta expression in human periodontal ligament cells," *Journal of Periodontal Research,* vol. 49, no. 5, pp. 595-602, 2014.

14. H. Kanzaki, M. Chiba, Y. Shimizu et al., "Periodontal ligament cells under mechanical stress induce osteoclastogenesis by receptor activator of nuclear factor kappaB ligand up-regulation via prostaglandin E2 synthesis," *Journal of Bone and Mineral Research,* vol. 17, no. 2, pp. 210-20, 2002.

15. J. Kikuta, M. Yamaguchi, M. Shimizu et al., "Notch signaling induces root resorption via RANKL and IL-6 from hPDL cells," *Journal of Dental Research,* vol. 94, no. 1, pp. 140-7, 2015.

16. S. J. Kim, K. H. Park, Y. G. Park et al., "Compressive stress induced the up-regulation of M-CSF, RANKL, TNF-a expression and the down-regulation of OPG expression in PDL cells via the integrin-FAK pathway," *Archives of Oral Biology,* vol. 58, no. 6, pp. 707-16, 2013.

17. C. Kirschneck, P. Proff, M. Maurer et al., "Orthodontic forces add to nicotine-induced loss of periodontal bone : An in vivo and in vitro study," *Journal of Orofacial Orthopedics,* vol. 76, no. 3, pp. 195-212, 2015.

18. R. Kunii, M. Yamaguchi, Y. Tanimoto et al., "Role of interleukin-6 in orthodontically induced inflammatory root resorption in humans," *Korean Journal of Orthodontics,* vol. 43, no. 6, pp. 294-301, 2013.

19. S. Y. Lee, H. I. Yoo and S. H. Kim, "CCR5-CCL axis in PDL during orthodontic biophysical force application," *Journal of Dental Research,* vol. 94, no. 12, pp. 1715-23, 2015.

20. F. Liu, F. Wen, D. He et al., "Force-induced H_2_S by PDLSCs modifies osteoclastic activity during tooth movement," *Journal of Dental Research,* vol. 96, no. 6, pp. 694-702, 2017.

21. L. Liu, K. Igarashi, H. Kanzaki et al., "Clodronate inhibits PGE_2_ production in compressed periodontal ligament cells," *Journal of Dental Research,* vol. 85, no. 8, pp. 757-60, 2006.

22. P. Luckprom, K. Kanjanamekanant and P. Pavasant, "Role of connexin43 hemichannels in mechanical stress-induced ATP release in human periodontal ligament cells," *Journal of Periodontal Research,* vol. 46, no. 5, pp. 607-15, 2011.

23. K. Mayahara, Y. Kobayashi, K. Takimoto et al., "Aging stimulates cyclooxygenase-2 expression and prostaglandin E_2_ production in human periodontal ligament cells after the application of compressive force," *Journal of Periodontal Research,* vol. 42, no. 1, pp. 8-14, 2007.

24. K. Mayahara, A. Yamaguchi, M. Sakaguchi et al., "Effect of Ga-Al-As laser irradiation on *COX-2* and *cPLA_2_-a* expression in compressed human periodontal ligament cells," *Lasers in Surgery and Medicine,* vol. 42, no. 6, pp. 489-93, 2010.

25. M. Mitsuhashi, M. Yamaguchi, T. Kojima et al., "Effects of HSP70 on the compression force-induced TNF-a and RANKL expression in human periodontal ligament cells," *Inflammation Research,* vol. 60, no. 2, pp. 187-94, 2011.

26. R. Nakajima, M. Yamaguchi, T. Kojima et al., "Effects of compression force on fibroblast growth factor-2 and receptor activator of nuclear factor kappa B ligand production by periodontal ligament cells *in vitro*," *Journal of Periodontal Research,* vol. 43, no. 2, pp. 168-73, 2008.

27. Y. Nishijima, M. Yamaguchi, T. Kojima et al., "Levels of RANKL and OPG in gingival crevicular fluid during orthodontic tooth movement and effect of compression force on releases from periodontal ligament cells *in vitro*," *Orthodontics and Craniofacial Research,* vol. 9, no. 2, pp. 63-70, 2006.

28. S. Premaraj, I. Souza and T. Premaraj, "Mechanical loading activates β-catenin signaling in periodontal ligament cells," *Angle Orthodontist,* vol. 81, no. 4, pp. 592-9, 2011.

29. S. Premaraj, I. Souza and T. Premaraj, "Focal adhesion kinase mediates β-catenin signaling in periodontal ligament cells," *Biochemical and Biophysical Research Communications,* vol. 439, no. 4, pp. 487-92, 2013.

30. P. Proff, C. Reicheneder, A. Faltermeier et al., "Effects of mechanical and bacterial stressors on cytokine and growth-factor expression in periodontal ligament cells," *Journal of Orofacial Orthopedics,* vol. 75, no. 3, pp. 191-202, 2014.

31. P. Römer, J. Köstler, V. Koretsi et al., "Endotoxins potentiate COX-2 and RANKL expression in compressed PDL cells," *Clinical Oral Investigations,* vol. 17, no. 9, pp. 2041-8, 2013.

32. P. Tripuwabhrut, M. Mustafa, C. G. Gjerde et al., "Effect of compressive force on human osteoblast-like cells and bone remodelling: an *in vitro* study," *Archives of Oral Biology,* vol. 58, no. 7, pp. 826-36, 2013.

33. P. Tripuwabhrut, K. Mustafa, P. Brudvik et al., "Initial responses of osteoblasts derived from human alveolar bone to various compressive forces," *European Journal of Oral Sciences,* vol. 120, no. 4, pp. 311-8, 2012.

34. M. Wolf, S. Lossdörfer, K. Küpper et al., "Regulation of high mobility group box protein 1 expression following mechanical loading by orthodontic forces *in vitro* and *in vivo*," *European Journal of Orthodontics,* vol. 36, no. 6, pp. 624-31, 2014.

35. M. Wolf, S. Lossdörfer, R. Craveiro et al., "Regulation of macrophage migration and activity by high-mobility group box 1 protein released from periodontal ligament cells during orthodontically induced periodontal repair: an in vitro and in vivo experimental study," *Journal of Orofacial Orthopedics,* vol. 74, no. 5, pp. 420-34, 2013.

36. S. Wongkhantee, T. Yongchaitrakul and P. Pavasant, "Mechanical stress induces osteopontin expression in human periodontal ligament cells through rho kinase," *Journal of Periodontology,* vol. 78, no. 6, pp. 1113-9, 2007.

37. K. Yamada, M. Yamaguchi, M. Asano et al., "Th17-cells in atopic dermatitis stimulate orthodontic root resorption," *Oral Diseases,* vol. 19, no. 7, pp. 683-93, 2013.

38. M. Yamaguchi, Y. Ozawa, A. Nogimura et al., "Cathepsins B and L increased during response of periodontal ligament cells to mechanical stress in vitro," *Connective Tissue Research,* vol. 45, no. 3, pp. 181-9, 2004.

39. M. Yamaguchi, N. Aihara, T. Kojima et al., "RANKL increase in compressed periodontal ligament cells from root resorption," *Journal of Dental Research,* vol. 85, no. 8, pp. 751-6, 2006.

40. Y. Zhang, X. Kou, N. Jiang et al., "Effect of intraoral mechanical stress application on the expression of a force-responsive prognostic marker associated with system disease progression," *Journal of Dentistry,* vol. 57, pp. 57-65, 2017.
