# Supplementary material for: In Vitro Weight-Loaded Cell Models for Understanding Mechanodependent Molecular Pathways Involved in Orthodontic Tooth Movement: A Systematic Review
Source: Stem Cells Int. 2018 Jul 31;2018:3208285. doi: 10.1155/2018/3208285 (PMC6091372; doi:10.1155/2018/3208285)
Supplement: Supplementary 3 — Studies applying the 2D weight approach on human and nonhuman cells and cell lines not included in Supplement 2 (i.e., human primary cells from the orofacial region). For each gene or metabolite force magnitude and force duration, the change in gene expression or substance secretion (increase, decrease, and no change) and the techniques applied are given. [file 3208285.f3.docx]

# Supplement 3. Studies applying the 2D weight approach on human and non-human cells and cell lines not included in Table 1. For each gene or metabolite force magnitude and force duration, the change in gene expression or substance secretion (increase, decrease, no change), and the techniques for analysis applied are given.

| **Reference** | **Cell type (species) ^a^** | **Gene symbol or metabolite** | **Examined force applied** | | **Gene expression ^c,d^**  **(Increase/ decrease/ no change)** | **Substance secretion ^d,e^**  **(Increase/ decrease/ no change)** |
| --- | --- | --- | --- | --- | --- | --- |
|  |  |  | **Magnitude [g/cm^2^]^b^** | **Duration [h]** |  |  |
| Goga et al. 2006 [1] | MG63 (*H. s.*) | *CASP3* | 2.0; 4.0 N/cm^2^ | 12; 24 | n. r. | Increase |
| Hayakawa et al. 2015 [2] | RAW264.7 (*M. m.*) | *Nfatc1* | 0.114; 0.215; 0.301; 0.387; 0.53^++^ | 1; 3; 6; 12; 24 | Increase (qPCR: GAPDH) | n. r. |
|  |  | *Tnfsf11* | 0.114; 0.215; 0.301; 0.387; 0.53^++^ | 1; 3; 6; 12; 24 | Increase (qPCR: GAPDH) | n. r. |
|  |  | *Tnfrsf11a* | 0.114; 0.215; 0.301; 0.387; 0.53^++^ | 1; 3; 6; 12; 24 | Increase (qPCR: GAPDH) | n. r. |
|  |  | *Ctsk* | 0.114; 0.215; 0.301; 0.387; 0.53^++^ | 1; 3; 6; 12; 24 | Increase (qPCR: GAPDH) | n. r. |
|  |  | *Clcn7* | 0.114; 0.215; 0.301; 0.387; 0.53^++^ | 1; 3; 6; 12; 24 | Increase (qPCR: GAPDH) | n. r. |
|  |  | *Mmp9* | 0.114; 0.215; 0.301; 0.387; 0.53^++^ | 1; 3; 6; 12; 24 | Increase (qPCR: GAPDH) | n. r. |
|  |  | *Tcirg1* | 0.114; 0.215; 0.301; 0.387; 0.53^++^ | 1; 3; 6; 12; 24 | Increase (qPCR: GAPDH) | n. r. |
|  |  | *Dcstamp* | 0.114; 0.215; 0.301; 0.387; 0.53^++^ | 1; 3; 6; 12; 24 | Increase (qPCR: GAPDH) | n. r. |
|  |  | *Ocstamp* | 0.114; 0.215; 0.301; 0.387; 0.53^++^ | 1; 3; 6; 12; 24 | Increase (qPCR: GAPDH) | n. r. |
|  |  | *Itgav* | 0.114; 0.215; 0.301; 0.387; 0.53^++^ | 1; 3; 6; 12; 24 | Increase (qPCR: GAPDH) | n. r. |
|  |  | *Itgb3* | 0.114; 0.215; 0.301; 0.387; 0.53^++^ | 1; 3; 6; 12; 24 | Increase (qPCR: GAPDH) | n. r. |
| Hoshina et al. 2004 [3] | Bone marrow derived osteoblasts (*R. n*.) | *Spp1* | 0.9 | 12; 24; 72 | No change (qPCR: GAPDH) | n. r. |
|  |  | *Bglap* | 0.9 | 12; 24; 72 | Decrease (qPCR: GAPDH) | n. r. |
|  |  | *Alpl* | 0.9 | 12; 24; 72 | n. r. | No change (activity) |
| Inubushi et al. 2014 [4] | ST-2 (*M. m.*) | *Tnf* | 0.5 | 2; 24 | Decrease (qPCR: 18S) | n. r. |
|  |  | *Ptgs2* | 0.5 | 2; 24 | Increase (qPCR: 18S) | n. r. |
|  |  | *Tnfsf11* | 0.5 | 2; 24 | Increase (qPCR: 18S) | n. r. |
|  |  | *Tnfrsf11b* | 0.5 | 2; 24 | Decrease (qPCR: 18S) | n. r. |
| Koyama et al. 2008 [5] | Saos-2 (*H. s.*) | *IL1B* | 0.5, 1.0, 2.0; 3.0 | 1; 3; 6; 9; 12; 24;  ELISA: 24 | Increase (qPCR: GAPDH) | Increase (ELISA) |
|  |  | *IL6* | 0.5, 1.0, 2.0; 3.0 | 1; 3; 6; 9; 12; 24;  ELISA: 24 | Increase (qPCR: GAPDH) | Increase (ELISA) |
|  |  | *IL8 (CXCL8)* | 0.5, 1.0, 2.0; 3.0 | 1; 3; 6; 9; 12; 24;  ELISA: 24 | No change (qPCR: GAPDH) | No change (ELISA) |
|  |  | *IL11* | 0.5, 1.0, 2.0; 3.0 | 1; 3; 6; 9; 12; 24;  ELISA: 24 | Increase (qPCR: GAPDH) | Increase (ELISA) |
|  |  | *TNF* | 0.5, 1.0, 2.0; 3.0 | 1; 3; 6; 9; 12; 24;  ELISA: 24 | Increase (qPCR: GAPDH) | Increase (ELISA) |
|  |  | *IL1R1* | 0.5, 1.0, 2.0; 3.0 | 1; 3; 6; 9; 12; 24; | Increase (qPCR: GAPDH) | n. r. |
|  |  | *IL6R* | 0.5, 1.0, 2.0; 3.0 | 1; 3; 6; 9; 12; 24; | Increase (qPCR: GAPDH) | n. r. |
|  |  | *CXCR1* | 0.5, 1.0, 2.0; 3.0 | 1; 3; 6; 9; 12; 24; | Increase (qPCR: GAPDH) | n. r. |
|  |  | *IL11RA* | 0.5, 1.0, 2.0; 3.0 | 1; 3; 6; 9; 12; 24; | No change (qPCR: GAPDH) | n. r. |
|  |  | *TNFRSF1A* | 0.5, 1.0, 2.0; 3.0 | 1; 3; 6; 9; 12; 24; | No change (qPCR: GAPDH) | n. r. |
| Matsunaga et al. 2016 [6] | Cementoblast cell line (HCEM-SV40) (*H.s.*) | *RUNX2* | 0.25 gf/cm^2^ | 12 | Decrease^+^ (qPCR: GAPDH) | n. r. |
|  |  | *ALPL* | 0.25 gf/cm^2^ | 12 | Decrease^+^ (qPCR: GAPDH) | n. r. |
|  |  | *WNT5A* | 0.25 gf/cm^2^ | 12 | Decrease^+^ (qPCR: GAPDH) | n. r. |
|  |  | *SPON1* | 0.25 gf/cm^2^ | 12 | Decrease^+^ (qPCR: GAPDH) | n. r. |
| Mitsui et al. 2005 [7] | Saos-2 (*H.s.*) | *PTGS2* | 1.0 | 1; 3; 6; 9; 12; 24 | Increase (qPCR: GAPDH) | n. r. |
|  |  | *IBSP* | 0.5, 1.0, 2.0; 3.0 WB: 1.0 | 1; 3; 6; 9; 12; 24 | Increase (qPCR: GAPDH) | Increase (WB) |
|  |  | *SPP1* | 1.0 | 1; 3; 6; 9; 12; 24 | Increase followed by decrease (qPCR: GAPDH) | Increase (WB) |
|  |  | PGE_2_ | 0.5, 1.0, 2.0; 3.0 | 1; 3; 6; 9; 12; 24 | n. a. | Increase (ELISA) |
| Mitsui et al. 2006 [8] | Saos-2 (*H.s.*) | *MMP1* | 0.5, 1.0, 2.0; 3.0; ELISA: 1.0 | 1; 3; 6; 9; 12; 24 | Increase (qPCR: GAPDH) | Increase (ELISA) |
|  |  | *MMP2* | 0.5, 1.0, 2.0; 3.0; ELISA: 1.0 | 1; 3; 6; 9; 12; 24 | Increase (qPCR: GAPDH) | Increase (ELISA) |
|  |  | *MMP3* | 0.5, 1.0, 2.0; 3.0; ELISA: 1.0 | 1; 3; 6; 9; 12; 24 | Increase (qPCR: GAPDH) | Increase (ELISA) |
|  |  | *MMP13* | 0.5, 1.0, 2.0; 3.0; ELISA: 1.0 | 1; 3; 6; 9; 12; 24 | Increase (qPCR: GAPDH) | Increase (ELISA) |
|  |  | *MMP14* | 0.5, 1.0, 2.0; 3.0; ELISA: 1.0 | 1; 3; 6; 9; 12; 24 | Increase (qPCR: GAPDH) | Increase (ELISA) |
|  |  | *TIMP1* | 0.5, 1.0, 2.0; 3.0; ELISA: 1.0 | 1; 3; 6; 9; 12; 24 | Increase (qPCR: GAPDH) | Increase (ELISA) |
|  |  | *TIMP2* | 0.5, 1.0, 2.0; 3.0; ELISA: 1.0 | 1; 3; 6; 9; 12; 24 | Increase (qPCR: GAPDH) | Increase (ELISA) |
|  |  | *TIMP3* | 0.5, 1.0, 2.0; 3.0; ELISA: 1.0 | 1; 3; 6; 9; 12; 24 | Increase (qPCR: GAPDH) | Increase (ELISA) |
|  |  | *TIMP4* | 0.5, 1.0, 2.0; 3.0; ELISA: 1.0 | 1; 3; 6; 9; 12; 24 | Increase (qPCR: GAPDH) | Increase (ELISA) |
|  |  | *PLAT* | 0.5, 1.0, 2.0; 3.0; ELISA: 1.0 | 1; 3; 6; 9; 12; 24 | Increase (qPCR: GAPDH) | Increase (ELISA) |
|  |  | *PLAU* | 0.5, 1.0, 2.0; 3.0; ELISA: 1.0 | 1; 3; 6; 9; 12; 24 | Increase (qPCR: GAPDH) | Increase (ELISA) |
|  |  | *SERPINE1* | 0.5, 1.0, 2.0; 3.0; ELISA: 1.0 | 1; 3; 6; 9; 12; 24 | Increase (qPCR: GAPDH) | Increase (ELISA) |
| Mitsui et al. 2006 [9] | Saos-2 (*H.s.*) | *BMP2* | 0.5, 1.0, 2.0; 3.0 | 1; 3; 6; 9; 12; 24; WB: 2 | Increase (qPCR: GAPDH) | Increase (WB) |
|  |  | *BMP4* | 0.5, 1.0, 2.0, or 3.0 | 9 | Increase (qPCR: GAPDH) | Increase (WB) |
|  |  | *BMP6* | 0.5, 1.0, 2.0, or 3.0 | 9 | Increase (qPCR: GAPDH) | Increase (WB) |
|  |  | *BMP7* | 0.5, 1.0, 2.0, or 3.0 | 9 | Increase (qPCR: GAPDH) | Increase (WB) |
|  |  | *BMPR1A* | 0.5, 1.0, 2.0, or 3.0 | 9 | Increase (qPCR: GAPDH) | n. r. |
|  |  | *BMPR1B* | 0.5, 1.0, 2.0, or 3.0 | 9 | Increase (qPCR: GAPDH) | n. r. |
|  |  | *ACVR1* | 0.5, 1.0, 2.0, or 3.0 | 9 | Increase (qPCR: GAPDH) | n. r. |
|  |  | *BMPR2* | 0.5, 1.0, 2.0, or 3.0 | 9 | Increase (qPCR: GAPDH) | n. r. |
|  |  | *ACVR2A* | 0.5, 1.0, 2.0, or 3.0 | 9 | Increase (qPCR: GAPDH) | n. r. |
|  |  | *ACVR2B* | 0.5, 1.0, 2.0, or 3.0 | 9 | Increase (qPCR: GAPDH) | n. r. |
|  |  | *CHRD* | 0.5, 1.0, 2.0, or 3.0 | 9 | Decrease followed by increase (qPCR: GAPDH) | n. r. |
|  |  | *GREM1* | 0.5, 1.0, 2.0, or 3.0 | 9 | Decrease followed by increase (qPCR: GAPDH) | Decrease followed by increase (WB) |
|  |  | *FST* | 0.5, 1.0, 2.0, or 3.0 | 9 | Decrease followed by no change (qPCR: GAPDH) | Decrease followed by no change (WB) |
|  |  | *NOG* | 0.5, 1.0, 2.0, or 3.0 | 9 | Decrease followed by increase (qPCR: GAPDH) | Decrease followed by increase (WB) |
|  |  | *RUNX2* | 0.5, 1.0, 2.0, or 3.0 | 9 | Increase (qPCR: GAPDH) | n. r. |
|  |  | *SP7* | 0.5, 1.0, 2.0, or 3.0 | 9 | Increase (qPCR: GAPDH) | n. r. |
|  |  | *ZNF354C* | 0.5, 1.0, 2.0, or 3.0 | 9 | Increase (qPCR: GAPDH) | n. r. |
|  |  | *SMAD1* | 1.0 | 9 | n. d. | Increase p-Smad1 (WB) |
| Rego et al. 2011 [10] | OCCM-30 (*M.m.*) | *Bmp2* | 0.2 kPa | 12 | Increase (qPCR: GAPDH) | n. r. |
|  |  | *Bglap2* | 0.2 kPa | 12 | Increase (qPCR: GAPDH) | n. r. |
|  |  | *Ptgs2* | 0.2 kPa | 1; 3; 6; 12; 24 | Increase (qPCR: GAPDH) | n. r. |
|  |  | *Tnfsf11* | 0.2 kPa | 12; 24 | Increase (qPCR: GAPDH) | n. r. |
|  |  | *Tnfrsf11b* | 0.2 kPa | 12; 24 | No change (qPCR: GAPDH) | n. r. |
|  |  | PGE_2_ | 0.2 kPa | 6; 12;24 | n. a. | Increase |
|  |  | *Ptger1* | 0.2 kPa | 1 | No change (qPCR: GAPDH) | n. r. |
|  |  | *Ptger2* | 0.2 kPa | 1 | No change (qPCR: GAPDH) | n. r. |
|  |  | *Ptger3* | 0.2 kPa | 1 | No change (qPCR: GAPDH) | n. r. |
|  |  | *Ptger4* | 0.2 kPa | 1 | No change (qPCR: GAPDH) | n. r. |
| Sanuki et al. 2010 [11] | MC3T3-E1 (*M.m.*) | *Ptgs2* | 1.0; 3.0 | 1; 3; 6; 9; 12; 24 | Increase (qPCR: GAPDH) | n. r. |
|  |  | *Csf1* | 1.0; 3.0 | 1; 3; 6; 9; 12; 24; ELISA: 24 | Increase (qPCR: GAPDH) | Increase (ELISA) |
|  |  | *Tnfsf11* | 1.0; 3.0 | 1; 3; 6; 9; 12; 24; ELISA: 24 | Increase (qPCR: GAPDH) | Increase (ELISA) |
|  |  | *Tnfrsf11b* | 1.0; 3.0 | 1; 3; 6; 9; 12; 24; ELISA: 24 | Decrease (qPCR: GAPDH) | Decrease (ELISA) |
|  |  | PGE_2_ | 1.0; 3.0 | 24 | n. a. | Increase (ELISA) |
| Takahashi et al. 2003 [12] | PDLC (*R. n*.) | *Mmp8* | 0.1; 0.2; 0.3 kPa | 72 | Decrease (sqPCR: GAPDH) | n. r. |
|  |  | *Mmp13* | 0.1; 0.2; 0.3 kPa | 72 | Decrease (sqPCR: GAPDH) | n. r. |
| Yanagisawa et al. 2007 [13] | C2C12 (*M.m.*) | *Runx2* | 0.25; 0.5, 1.0, 2.0 | 1; 3; 6; 9; 12; 24 (WB: 24) | Increase (qPCR: GAPDH) | Increase (WB) |
|  |  | *Msx2* | 0.25; 0.5, 1.0, 2.0 | 1; 3; 6; 9; 12; 24 (WB: 24) | Increase (qPCR: GAPDH) | Increase (WB) |
|  |  | *Dlx5* | 0.25; 0.5, 1.0, 2.0 | 1; 3; 6; 9; 12; 24 (WB: 24) | Increase (qPCR: GAPDH) | Increase (WB) |
|  |  | *Sp7* | 0.25; 0.5, 1.0, 2.0 | 1; 3; 6; 9; 12; 24 (WB: 24) | Increase (qPCR: GAPDH) | Increase (WB) |
|  |  | *Zfp354c* | 0.25; 0.5, 1.0, 2.0 | 1; 3; 6; 9; 12; 24 (WB: 24) | Increase (qPCR: GAPDH) | Increase (WB) |
|  |  | *Sox5* | 0.25; 0.5, 1.0, 2.0 | 1; 3; 6; 9; 12; 24 (WB: 24) | Increase (qPCR: GAPDH) | Increase (WB) |
|  |  | *Sox9* | 0.25; 0.5, 1.0, 2.0 | 1; 3; 6; 9; 12; 24 (WB: 24) | Increase (qPCR: GAPDH) | Increase (WB) |
|  |  | *Myod1* | 0.25; 0.5, 1.0, 2.0 | 1; 3; 6; 9; 12; 24 (WB: 24) | Increase (qPCR: GAPDH) | Increase (WB) |
|  |  | *Pparg* | 0.25; 0.5, 1.0, 2.0 | 1; 3; 6; 9; 12; 24 (WB: 24) | Increase (qPCR: GAPDH) | Increase (WB) |
|  |  | *p38-MAPK (*Antibody specificity not specified)* | 0.5 | WB/ELISA: 5, 10, 20, 30, 60 min | n. r. | Increased p-P38 MAPK (WB)  Increased p-P38 MAPK (ELISA) |
| Zhang et al. 2010 [14] | MC3T3-E1 (*M.m.*) | *Il17a* | 1.0; 2.0 | 1; 3; 6; 9; 12; 24 | Increase (qPCR: GAPDH) | n. r. |
|  |  | *Il17b* | 1.0; 2.0 | 1; 3; 6; 9; 12; 24 | Increase (qPCR: GAPDH) | n. r. |
|  |  | *Il17d* | 1.0; 2.0 | 1; 3; 6; 9; 12; 24 | Increase (qPCR: GAPDH) | n. r. |
|  |  | *Il17c* | 1.0; 2.0 | 1; 3; 6; 9; 12; 24 | Increase (qPCR: GAPDH) | n. r. |
|  |  | *Il25* | 1.0; 2.0 | 1; 3; 6; 9; 12; 24 | Increase (qPCR: GAPDH) | n. r. |
|  |  | *Il17f* | 1.0; 2.0 | 1; 3; 6; 9; 12; 24 | Increase (qPCR: GAPDH) | n. r. |
|  |  | *Il17ra* | 1.0; 2.0 | 1; 3; 6; 9; 12; 24 | Increase (qPCR: GAPDH) | n. r. |
|  |  | *Il17rb* | 1.0; 2.0 | 1; 3; 6; 9; 12; 24 | Increase (qPCR: GAPDH) | n. r. |
|  |  | *Il17rd* | 1.0; 2.0 | 1; 3; 6; 9; 12; 24 | Increase (qPCR: GAPDH) | n. r. |
|  |  | *Il17rc* | 1.0; 2.0 | 1; 3; 6; 9; 12; 24 | Increase (qPCR: GAPDH) | n. r. |
|  |  | *Il17re* | 1.0; 2.0 | 1; 3; 6; 9; 12; 24 | Increase (qPCR: GAPDH) | n. r. |
|  |  | *Il1a* | 1.0; 2.0 | 2 | n. r. | Increase (ELISA) |
|  |  | *Il6* | 1.0; 2.0 | 2 | n. r. | Increase (ELISA) |
| Zhang et al. 2017 [15] | OCCM-30 (*M.m.*) | *Piezo1* | 2.0 | 0; 3; 6; 9; 12; 24; | Decrease (qPCR: GAPDH) | Decrease (WB) |
|  |  | *Tnfrsf11b* | 2.0 | 0; 3; 6; 9; 12; 24; | Decrease (qPCR: GAPDH) | n. r. |
|  |  | *Spp1* | 2.0 | 0; 3; 6; 9; 12; 24; | Decrease (qPCR: GAPDH) | n. r. |
|  |  | *Bglap* | 2.0 | 0; 3; 6; 9; 12; 24; | Decrease (qPCR: GAPDH) | n. r. |
|  |  | *Hacd1* | 2.0 | 0; 3; 6; 9; 12; 24; | Decrease (qPCR: GAPDH) | n. r. |
| Zhou et al. 2013 [16] | U2OS (*H.s.*) | *RUNX2* | 1.0 | 1; 4; 8; 12; 24 | Decrease (qPCR: GAPDH) | n. r. |
|  |  | *BGLAP* | 1.0 | 1; 4; 8; 12; 24 | Decrease (qPCR: GAPDH) | n. r. |
|  |  | *ALPL* | 1.0 | 1; 4; 8; 12; 24 | Decrease (qPCR: GAPDH) | n. r. |
|  |  | *IBSP* | 1.0 | 1; 4; 8; 12; 24 | Increase (qPCR: GAPDH) | n. r. |
|  |  | *IL1B* | 1.0 | 1; 4; 8; 12; 24 | Increase (qPCR: GAPDH) | n. r. |
|  |  | *IL6* | 1.0 | 1; 4; 8; 12; 24 | Increase (qPCR: GAPDH) | n. r. |
|  |  | *PTGS2* | 1.0 | 1; 4; 8; 12; 24 | Increase (qPCR: GAPDH) | n. r. |

^a^ Origin of cells: *H. s*. – *H. sapiens*; *M. m*. – *M. musculus*; *R. n*. – *Rattus norvegicus*

^b +^Analysis was done 7, 14 or 21 days after WAB;  ^++^ Calculated according to information given in the respective study

^c^ qPCR – quantitative PCR (e.g. real time PCR); sqPCR – semi-quantitative PCR; followed by reference gene used

^d^ n. r. – not reported; n. a. – not applicable

^e^ ELISA – Enzyme linked immune absorbent assay; WB – western blot; IF – immunofluorescence; p-Smad1 – phosphorylated Smad1 (“similar to mothers against decapentaplegic 1”); p-P38 MAPK – phosphorylated P38 mitogen-activated protein kinases

References:

1. Y. Goga, M. Chiba, Y. Shimizu et al., "Compressive force induces osteoblast apoptosis *via* caspase-8," *Journal of Dental Research,* vol. 85, no. 3, pp. 240-4, 2006.

2. T. Hayakawa, Y. Yoshimura, T. Kikuiri et al., "Optimal compressive force accelerates osteoclastogenesis in RAW264.7 cells," *Mol Med Rep,* vol. 12, no. 4, pp. 5879-85, 2015.

3. S. Hoshina, K. Matsuzaka, Y. Motoyoshi et al., "Osteoblast-like cell behavior of rat bone marrow under continuous compressive force *in vitro*," *Biomedical Research,* vol. 25, no. 3, pp. 109-117, 2004.

4. T. Inubushi, A. Kawazoe, M. Miyauchi et al., "Lactoferrin inhibits infection-related osteoclastogenesis without interrupting compressive force-related osteoclastogenesis," *Archives of Oral Biology,* vol. 59, no. 2, pp. 226-32, 2014.

5. Y. Koyama, N. Mitsui, N. Suzuki et al., "Effect of compressive force on the expression of inflammatory cytokines and their receptors in osteoblastic Saos-2 cells," *Archives of Oral Biology,* vol. 53, no. 5, pp. 488-96, 2008.

6. K. Matsunaga, C. Ito, K. Nakakogawa et al., "Response to light compressive force in human cementoblasts *in vitro*," *Biomedical Research,* vol. 37, no. 5, pp. 293-298, 2016.

7. N. Mitsui, N. Suzuki, M. Maeno et al., "Optimal compressive force induces bone formation via increasing bone sialoprotein and prostaglandin E_2_ production appropriately," *Life Sciences,* vol. 77, no. 25, pp. 3168-82, 2005.

8. N. Mitsui, N. Suzuki, Y. Koyama et al., "Effect of compressive force on the expression of MMPs, PAs, and their inhibitors in osteoblastic Saos-2 cells," *Life Sciences,* vol. 79, no. 6, pp. 575-83, 2006.

9. N. Mitsui, N. Suzuki, M. Maeno et al., "Optimal compressive force induces bone formation via increasing bone morphogenetic proteins production and decreasing their antagonists production by Saos-2 cells," *Life Sciences,* vol. 78, no. 23, pp. 2697-706, 2006.

10. E. B. Rego, T. Inubushi, A. Kawazoe et al., "Effect of PGE_2_ induced by compressive and tensile stresses on cementoblast differentiation *in vitro*," *Archives of Oral Biology,* vol. 56, no. 11, pp. 1238-46, 2011.

11. R. Sanuki, C. Shionome, A. Kuwabara et al., "Compressive force induces osteoclast differentiation via prostaglandin E_2_ production in MC3T3-E1 cells," *Connective Tissue Research,* vol. 51, no. 2, pp. 150-8, 2010.

12. I. Takahashi, M. Nishimura, K. Onodera et al., "Expression of MMP-8 and MMP-13 genes in the periodontal ligament during tooth movement in rats," *Journal of Dental Research,* vol. 82, no. 8, pp. 646-51, 2003.

13. M. Yanagisawa, N. Suzuki, N. Mitsui et al., "Effects of compressive force on the differentiation of pluripotent mesenchymal cells," *Life Sciences,* vol. 81, no. 5, pp. 405-12, 2007.

14. F. Zhang, C. L. Wang, Y. Koyama et al., "Compressive force stimulates the gene expression of IL-17s and their receptors in MC3T3-E1 cells," *Connective Tissue Research,* vol. 51, no. 5, pp. 359-69, 2010.

15. Y. Y. Zhang, Y. P. Huang, H. X. Zhao et al., "Cementogenesis is inhibited under a mechanical static compressive force via *Piezo1*," *Angle Orthodontist,* vol. 87, no. 4, pp. 618-624, 2017.

16. S. Zhou, J. Zhang, H. Zheng et al., "Inhibition of mechanical stress-induced NF-κB promotes bone formation," *Oral Diseases,* vol. 19, no. 1, pp. 59-64, 2013.
