# Supplementary material for: In Vitro Weight-Loaded Cell Models for Understanding Mechanodependent Molecular Pathways Involved in Orthodontic Tooth Movement: A Systematic Review
Source: Stem Cells Int. 2018 Jul 31;2018:3208285. doi: 10.1155/2018/3208285 (PMC6091372; doi:10.1155/2018/3208285)
Supplement: Supplementary 4 — Studies applying the 3D weight approach on human and nonhuman cells and cell lines. For each gene or metabolite force magnitude and force duration, the change in gene expression or substance secretion (increase, decrease, and no change) and the techniques applied are given. [file 3208285.f4.docx]

# Supplement 4. Studies applying the 3D weight approach on human and non-human cells and cell lines. For each gene or metabolite force magnitude and force duration are given, the change in gene expression or substance secretion (increase, decrease, no change), and the techniques for analysis applied are given.

| **Reference** | **Cell type ^a^** | **Gene symbol or metabolite** | **Scaffold ^b^** | **Examined force applied** | | **Gene expression ^d,e^**  **(Increase/ decrease/ no change)** | **Substance secretion ^e,f^**  **(Increase/ decrease/ no change)** |
| --- | --- | --- | --- | --- | --- | --- | --- |
|  |  |  |  | **Magnitude [g/cm^2^]^c^** | **Duration [h]** |  |  |
| Santos de Araujo et al. 2014 [1] | hPDLC (exp) | F-actin (Triton X-100 insoluble fraction) | Collagen gel | 6.0 (IF/WB: 3.0) | 48 | n. r. | Decrease (WB)  Reversible inhibition of stress fibre formation (IF) |
|  |  | *RND3* | Collagen gel | 6.0 (IF/WB: 3.0) | 12; 24 | n. r. | Increase (WB) |
|  |  | *RHOA* | Collagen gel | 6.0 (IF/WB: 3.0) | 12; 24 | n. r. | No change (WB) |
|  |  | *RGS2* | Collagen gel | 6.0 (IF/WB: 3.0) | 12; 24 | n. r. | Increase (WB) |
| Santos de Araujo et al. 2007 [2] | hPLDF (?) | *RGS2* | Collagen gel | 6.0 | qPCR: 6; 12; 24  sqPCR: 6  WB: 12; 24; 48 | Increase (sqPCR & qPCR: GAPDH) | Increase (WB) |
|  |  | cAMP_i_ | Collagen gel | 6.0 | 1; 3; 6; 12 | n. r. | Increase (EIA) |
| de Araujo et al. 2007 [3] | hPDLC (exp) | PGE_2_ | Collagen gel | 6.0 | 3; 12; 24; 48; 72 | n. r. | Increase (EIA) |
|  |  | *PTGS2* | Collagen gel | 3.6; 6.0; 7.1; 9.5 | 1; 3; 6; 12; 24; 48; 72 | Increase (sqPCR: GAPDH) | n. r. |
|  |  | *HSPA5* | Collagen gel | 6.0 | 6; 12; 24 | Increase (qPCR: GAPDH) | n. r. |
|  |  | *IL6* | Collagen gel | 6.0 | 6; 12; 24 | Increase (qPCR: GAPDH) | n. r. |
|  |  | *RND3* | Collagen gel | 6.0 | 6; 12; 24 | Increase (qPCR: GAPDH) | n. r. |
|  |  | *IL1B* | Collagen gel | 6.0 | 6; 12; 24 | Increase (qPCR: GAPDH) | n. r. |
|  |  | *RCAN1* | Collagen gel | 6.0 | 6; 12; 24 | Increase (qPCR: GAPDH) | n. r. |
|  |  | *INP4A* | Collagen gel | 6.0 | 6; 12; 24 | Decrease (qPCR: GAPDH) | n. r. |
| Kaku et al. 2016 [4] | hPDLC (dig) | *TNFRSF11B* | Collagen gel | 0.5; 1.0; 2.0 | 12;24 | Increase (qPCR: GAPDH) | n. r. |
|  |  | *COL1A2* | Collagen gel | 0.5; 1.0; 2.0 | 12;24 | No change (qPCR: GAPDH) | n. r. |
|  |  | *LOX* | Collagen gel | 0.5; 1.0; 2.0 | 12;24 | Increase (qPCR: GAPDH) | n. r. |
|  |  | *PLOD1* | Collagen gel | 0.5; 1.0; 2.0 | 12;24 | No change (qPCR: GAPDH) | n. r. |
|  |  | *PLOD2* | Collagen gel | 0.5; 1.0; 2.0 | 12;24 | Increase (qPCR: GAPDH) | n. r. |
|  |  | *PLOD3* | Collagen gel | 0.5; 1.0; 2.0 | 12;24 | No change (qPCR: GAPDH) | n. r. |
| Kaneuji et al. 2011 [5] | MC3T3-E1 (*M. m.)* | *TNFRSF11B* | Collagen gel | 7.5; ELISA: 2.5; 7.5 | 24; ELISA: 48 | Increase (sqPCR: β-Actin) | Increase (ELISA) |
| Kang et al. 2013 [6] | hPDLC (dig) | *IL1B* | Collagen gel | 2.0 | 2;48 | Increase (qPCR: GAPDH) | n. r. |
|  |  | *TNF* | Collagen gel | 2.0 | 2;48 | Increase (qPCR: GAPDH) | n. r. |
|  |  | *TNFSF11* | Collagen gel | 2.0 | 2;48 | Increase (qPCR: GAPDH) | n. r. |
|  |  | *MMP3* | Collagen gel | 2.0 | 2;48 | Increase (qPCR: GAPDH) | n. r. |
|  |  | *MMP13* | Collagen gel | 2.0 | 2;48 | Increase (qPCR: GAPDH) | n. r. |
| Lee et al. 2007 [7] | hPDLC (?) | *ALPP* | Collagen gel | 1.76 | 2; 12  ELISA: 72 | Increase (qPCR: GAPDH) | Increase (ELISA) |
|  |  | *IL6* | Collagen gel | 1.76 | 2; 12  ELISA: 72 | Increase (qPCR: GAPDH) | Decrease (ELISA) |
|  |  | *CXCL8* | Collagen gel | 1.76 | 2; 12  ELISA: 72 | Increase (qPCR: GAPDH) | No change (ELISA) |
| Li et al. 2016 [8] | hPDLC (dig) | *TNFSF11* | PLGA | 25.0 | 6;24;72 | Increase (qPCR: GAPDH) | n. r. |
|  |  | *TNFRSF11B* | PLGA | 25.0 | 6;24;72 | Decrease (6h) followed by Increase (24,72h) (qPCR: GAPDH) | n. r. |
|  |  | PTGS2 | PLGA | 25.0 | 6;24;72 | Increase (qPCR: GAPDH) | n. r. |
|  |  | *IL1B* | PLGA | 25.0 | 6;24;72 | Increase (qPCR: GAPDH) | n. r. |
|  |  | *HIF1A* | PLGA | 25.0 | 6;24;72 | No change (qPCR: GAPDH) | n. r. |
|  |  | *VEGFA* | PLGA | 25.0 | 6;24;72 | Increase (qPCR: GAPDH) | n. r. |
| Li et al. 2013 [9] | hPDLC (dig) | *CCL20* | PLGA | 25.0 | 6; 24; 72 | Increase (qPCR: GAPDH) | n. r. |
|  |  | *STC1* | PLGA | 25.0 | 6; 24; 72 | Increase (qPCR: GAPDH) | n. r. |
|  |  | *IL1RN* | PLGA | 25.0 | 6; 24; 72 | Increase (6; 24h) followed by decrease(72h) (qPCR: GAPDH) | n. r. |
|  |  | *NOG* | PLGA | 25.0 | 6; 24; 72 | Increase (qPCR: GAPDH) | n. r. |
|  |  | *FGF7* | PLGA | 25.0 | 6; 24; 72 | Increase (qPCR: GAPDH) | n. r. |
|  |  | *FOS* | PLGA | 25.0 | 6; 24; 72 | Increase (qPCR: GAPDH) | n. r. |
|  |  | *MAP3K8* | PLGA | 25.0 | 6; 24; 72 | Decrease (6h) followed by increase (24; 72h) (qPCR: GAPDH) | n. r. |
|  |  | *JUN* | PLGA | 25.0 | 6; 24; 72 | Decrease (6h) followed by increase (24; 72h) (qPCR: GAPDH) | n. r. |
|  |  | *CDK1* | PLGA | 25.0 | 6; 24; 72 | Decrease (qPCR: GAPDH) | n. r. |
|  |  | *CCNA2* | PLGA | 25.0 | 6; 24; 72 | Decrease (qPCR: GAPDH) | n. r. |
|  |  | *KIF11* | PLGA | 25.0 | 6; 24; 72 | Decrease (qPCR: GAPDH) | n. r. |
|  |  | *KIF23* | PLGA | 25.0 | 6; 24; 72 | Decrease (qPCR: GAPDH) | n. r. |
|  |  | *CYR61* | PLGA | 25.0 | 6; 24; 72 | Decrease (6h) followed by increase (24; 72h) (qPCR: GAPDH) | n. r. |
|  |  | *COX1* | PLGA | 25.0 | 6; 24; 72 | Increase (qPCR: GAPDH) | n. r. |
|  |  | *PTGS2* | PLGA | 25.0 | 6; 24; 72 | Increase (qPCR: GAPDH) | n. r. |
| Li et al. 2016 [10] | hPDLC (dig) | *TNFSF11* | PLGA | 5.0; 15.0; 25.0 | 6; 24; 72 | Increase (qPCR: GAPDH) | Decrease (ELISA) |
|  |  | PTGS2 | PLGA | 5.0; 15.0; 25.0 | 6; 24; 72 | Increase (qPCR: GAPDH) | n. r. |
|  |  | *PTHLH* | PLGA | 5.0; 15.0; 25.0 | 6; 24; 72 | Increase (qPCR: GAPDH) | Increase (ELISA) |
|  |  | *IL11* | PLGA | 5.0; 15.0; 25.0 | 6; 24; 72 | Increase (qPCR: GAPDH) | Increase (ELISA) |
|  |  | *TNFRSF11B* | PLGA | 5.0; 15.0; 25.0 | 6; 24; 72 | Increase (qPCR: GAPDH) | Decrease followed by Increase (ELISA) |
|  |  | PGE_2_ | PLGA | 5.0; 15.0; 25.0 | 6; 24; 72 | n. a. | Increase (ELISA) |
| Li et al. 2011 [11] | hPDLC (exp) | *TNFSF11* | PLGA | 5; 15; 25; 35 | 6; 24; 72 | Increase (6; 24h) followed by decrease (72h) (qPCR: GAPDH) | n. r. |
|  |  | *PTGS2* | PLGA | 5; 15; 25; 35 | 6 | Increase (qPCR: GAPDH) | n. r. |
|  |  | *TNFRSF11B* | PLGA | 25 | 6; 24; 72 | Decrease (6h) followed by increase (24; 72h) (qPCR: GAPDH) | n. r. |
|  |  | *IL1B* | PLGA | 25 | 6; 24; 72 | No change (qPCR: GAPDH) | n. r. |
|  |  | *CXCL8* | PLGA | 25 | 6; 24; 72 | Increase (qPCR: GAPDH) | n. r. |
|  |  | *IL11* | PLGA | 25 | 6; 24; 72 | Increase (qPCR: GAPDH) | n. r. |
|  |  | *FGF2* | PLGA | 25 | 6; 24; 72 | Increase (qPCR: GAPDH) | n. r. |
|  |  | *PTHLH* | PLGA | 25 | 6; 24; 72 | Increase (qPCR: GAPDH) | n. r. |
|  |  | *RUNX2* | PLGA | 25 | 6; 24; 72 | No change (qPCR: GAPDH) | n. r. |
|  |  | *BMP2* | PLGA | 25 | 6; 24; 72 | Increase (qPCR: GAPDH) | n. r. |
|  |  | *POSTN* | PLGA | 25 | 6; 24; 72 | Decrease (qPCR: GAPDH) | n. r. |
|  | hGF (exp) | *CXCL8* | PLGA | 25 | 6; 24; 72 | Increase (qPCR: GAPDH) | n. r. |
|  |  | *TNFSF11* | PLGA | 25 | 6; 24; 72 | No change (qPCR: GAPDH) | n. r. |
|  |  | *TNFRSF11B* | PLGA | 25 | 6; 24; 72 | No change (qPCR: GAPDH) | n. r. |
|  |  | *PTHLH* | PLGA | 25 | 6; 24; 72 | No change (qPCR: GAPDH) | n. r. |
|  |  | *IL11* | PLGA | 25 | 6; 24; 72 | No change (qPCR: GAPDH) | n. r. |
|  |  | *FGF2* | PLGA | 25 | 6; 24; 72 | No change (qPCR: GAPDH) | n. r. |
| Liao et al. 2016 [12] | hPDLC ($$) | *TNFSF11* | PLLA modif. | 5.0; 15.0; 25.0; 35.0 | 1d; 3d; 7d; 14d | Increase (qPCR: GAPDH) | n. r. |
|  |  | *BMP2* | PLLA modif. | 5.0; 15.0; 25.0; 35.0 | 1d; 3d; 7d; 14d | Increase (qPCR: GAPDH) | n. r. |
|  |  | *ASPN* | PLLA modif. | 5.0; 15.0; 25.0; 35.0 | 1d; 3d; 7d; 14d | Increase (qPCR: GAPDH) | n. r. |
|  |  | *ALPP* | PLLA modif. | 5.0; 15.0; 25.0; 35.0 | 1d; 3d; 7d; 14d | Increase (qPCR: GAPDH) | n. r. |
|  |  | *TNFRSF11B* | PLLA modif. | 5.0; 15.0; 25.0; 35.0 | 1d; 3d; 7d; 14d | No change (qPCR: GAPDH) | n. r. |
|  |  | *COL1A1* | PLLA modif. | 5.0; 15.0; 25.0; 35.0 | 1d; 3d; 7d; 14d | Decrease (qPCR: GAPDH) | n. r. |
|  |  | *FGF2* | PLLA modif. | 5.0; 15.0; 25.0; 35.0 | 1d; 3d; 7d; 14d | No change (qPCR: GAPDH) | n. r. |
| Shen et al. 2017 [13] | MC3T3-E1/SC14 | *Runx2* | Collagen gel | 0; 1; 2; 3; 4; 5 | 24 | Increase (qPCR: Bactn) | Increase (WB) |
|  |  | *Alp* | Collagen gel | 0; 1; 2; 3; 4; 5 | 24 | Increase (qPCR: Bactn) | Increase (WB);  Increase (Activity) |
|  |  | *Ocn* | Collagen gel | 0; 1; 2; 3; 4; 5 | 24 | Increase (qPCR: Bactn) | Increase (WB) |
|  |  | *Rankl* | Collagen gel | 0; 1; 2; 3; 4; 5 | 24 | Increase (qPCR: Bactn) | Not detectable (ELISA) |
|  |  | *Opg* | Collagen gel | 0; 1; 2; 3; 4; 5 | 24 | Increase (qPCR: Bactn) | Increase (ELISA) |
|  | mOB | *Runx2* | Collagen gel | 0; 1; 2; 3; 4; 5 | 24 | Increase (qPCR: Bactn) | n. r. |
|  |  | *Alp* | Collagen gel | 0; 1; 2; 3; 4; 5 | 24 | Increase (qPCR: Bactn) | Increase (Activity) |
|  |  | *Ocn* | Collagen gel | 0; 1; 2; 3; 4; 5 | 24 | Increase (qPCR: Bactn) | n. r. |
|  |  | *Rankl* | Collagen gel | 0; 1; 2; 3; 4; 5 | 24 | Increase (qPCR: Bactn) | n. r. |
|  |  | *Opg* | Collagen gel | 0; 1; 2; 3; 4; 5 | 24 | Increase (qPCR: Bactn) | n. r. |
|  |  | *Ocn* | Collagen gel | 0; 1; 2; 3; 4; 5 | 24 | Increase (qPCR: Bactn) | n. r. |
| Jianru et al. 2015 [14] | hPDLC (dig) | *TNFSF11* | PLGA | 25.0 | 3; 6; 12 (WB: 12) | Increase (qPCR: GAPDH) | Increase (WB) |
|  |  | *TNFRSF11B* | PLGA | 25.0 | 3; 6; 12 (WB: 12) | Decrease (3h) followed by increase (6,12h) (qPCR: GAPDH) | Increase (WB) |
|  |  | *NFATC2* | PLGA | 25.0 | 3; 6; 12 | Increase (qPCR: GAPDH) | n. r. |
| Yi et al. 2016 [15] | hPDLC (exp) | *TNFSF11* | PLGA | 25.0 | 24 | Increase (qPCR: GAPDH) | Increase (WB) |
|  |  | *TNFRSF11B* | PLGA | 25.0 | 24 | Decrease (qPCR: GAPDH) | No change (WB) |
|  |  | *PTHLH* | PLGA | 25.0 | 24 | Increase (qPCR: GAPDH) | n. r. |
|  |  | *PTGS2* | PLGA | 25.0 | 24 | Increase (qPCR: GAPDH) | Increase (WB) |
|  |  | *CXCL8* | PLGA | 25.0 | 24 | Increase (qPCR: GAPDH) | n. r. |
|  |  | *IL11* | PLGA | 25.0 | 24 | Increase (qPCR: GAPDH) | n. r. |
|  |  | PGE_2_ | PLGA | 25.0 | 24 | n. a. | Increase (ELISA) |

^a^ hPDLC (exp) – hPDLC, isolated with explant method; hPDLC (dig) – hPDLC, isolated with digestion method, hPDLC (?) – hPDLC, isolation method not given; hPDLC ($$) – hPDLC from commercial sources; hOB – human osteoblasts; hOBMC – human oral bone marrow cells; hGF – human gingival fibroblasts

Origin of non-human cells: *M. m*. – *M. musculus*

^b^ PLGA – Poly lactic-co-glycolic acid; PLLA modif. – Hydrophilically modified poly-L-lactide acid matrix

^c^ IF – immunofluorescence; WB – western blot; ELISA - Enzyme linked immune absorbent assay;

^d^ qPCR – quantitative polymerase chain reaction (e.g. real time PCR); sqPCR – semi-quantitative polymerase chain reaction; followed by reference gene used

^e^ n. r. – not reported; n. a. – not applicable

^f^ ELISA – Enzyme linked immune absorbent assay; WB – western blot; IF – immunofluorescence

References

1. R. M. Santos de Araujo, Y. Oba, S. Kuroda et al., "RhoE regulates actin cytoskeleton organization in human periodontal ligament cells under mechanical stress," *Archives of Oral Biology,* vol. 59, no. 2, pp. 187-92, 2014.

2. R. M. Santos de Araujo, Y. Oba and K. Moriyama, "Role of regulator of G-protein signaling 2 (RGS2) in periodontal ligament cells under mechanical stress," *Cell Biochemistry and Function,* vol. 25, no. 6, pp. 753-8, 2007.

3. R. M. de Araujo, Y. Oba and K. Moriyama, "Identification of genes related to mechanical stress in human periodontal ligament cells using microarray analysis," *Journal of Periodontal Research,* vol. 42, no. 1, pp. 15-22, 2007.

4. M. Kaku, J. M. Rosales Rocabado, M. Kitami et al., "Mechanical loading stimulates expression of collagen cross-linking associated enzymes in periodontal ligament," *Journal of Cellular Physiology,* vol. 231, no. 4, pp. 926-33, 2016.

5. T. Kaneuji, W. Ariyoshi, T. Okinaga et al., "Mechanisms involved in regulation of osteoclastic differentiation by mechanical stress-loaded osteoblasts," *Biochemical and Biophysical Research Communications,* vol. 408, no. 1, pp. 103-9, 2011.

6. K. L. Kang, S. W. Lee, Y. S. Ahn et al., "Bioinformatic analysis of responsive genes in two-dimension and three-dimension cultured human periodontal ligament cells subjected to compressive stress," *Journal of Periodontal Research,* vol. 48, no. 1, pp. 87-97, 2013.

7. Y. H. Lee, D. S. Nahm, Y. K. Jung et al., "Differential gene expression of periodontal ligament cells after loading of static compressive force," *Journal of Periodontology,* vol. 78, no. 3, pp. 446-52, 2007.

8. M. L. Li, J. Yi, Y. Yang et al., "Compression and hypoxia play independent roles while having combinative effects in the osteoclastogenesis induced by periodontal ligament cells," *Angle Orthodontist,* vol. 86, no. 1, pp. 66-73, 2016.

9. Y. Li, M. Li, L. Tan et al., "Analysis of time-course gene expression profiles of a periodontal ligament tissue model under compression," *Archives of Oral Biology,* vol. 58, no. 5, pp. 511-22, 2013.

10. M. Li, J. Yi, Y. Yang et al., "Investigation of optimal orthodontic force at the cellular level through three-dimensionally cultured periodontal ligament cells," *European Journal of Orthodontics,* vol. 38, no. 4, pp. 366-72, 2016.

11. Y. Li, W. Zheng, J. S. Liu et al., "Expression of osteoclastogenesis inducers in a tissue model of periodontal ligament under compression," *Journal of Dental Research,* vol. 90, no. 1, pp. 115-20, 2011.

12. W. Liao, M. Okada, K. Inami et al., "Cell survival and gene expression under compressive stress in a three-dimensional in vitro human periodontal ligament-like tissue model," *Cytotechnology,* vol. 68, no. 2, pp. 249-60, 2016.

13. X. Q. Shen, Y. M. Geng, P. Liu et al., "Magnitude-dependent response of osteoblasts regulated by compressive stress," *Scientific Reports,* vol. 7, pp. 44925, 2017.

14. Y. I. Jianru, L. I. MeiLe, Y. Yang et al., "Static compression regulates OPG expression in periodontal ligament cells via the CAMK II pathway," *J Appl Oral Sci,* vol. 23, no. 6, pp. 549-54, 2015.

15. J. Yi, B. Yan, M. Li et al., "Caffeine may enhance orthodontic tooth movement through increasing osteoclastogenesis induced by periodontal ligament cells under compression," *Archives of Oral Biology,* vol. 64, pp. 51-60, 2016.
